# Thiamine hydrochloride (VB_1_) in aqueous media catalyzed the synthesis of polysubstituted quinolines *via* a one-pot strategy: a combined experimental and theoretical investigation†

**DOI:** 10.1039/d5ra01461a

**Published:** 2025-04-24

**Authors:** Mina Hajipour, Hossein Mehrabi, Hamid Reza Masoodi

**Affiliations:** a Department of Chemistry, Vali-e-Asr University of Rafsanjan Rafsanjan Iran h.mehrabi@vru.ac.ir

## Abstract

In this work, an efficient one-pot three-component reaction of 2-cyano-*N*-methylacetamide, arylglyoxals, and arylamines in the presence of thiamine hydrochloride in H_2_O under reflux conditions was designed for the synthesis of 4-amino-2-benzoylquinoline-3-carboxamide. In this protocol, various synthetic methods such as Knoevenagel/Michael/cyclization cascade reactions were used to introduce different functional groups, such as amino and carboxamide groups, on the quinoline ring system in a single step. In addition to operational simplicity and absence of tedious separation procedures, this method offered the advantages of catalyst reusability and high product yields. Characterization techniques such as nuclear magnetic resonance spectroscopy, infrared spectroscopy, and CHN analysis were used to confirm the structure and purity of the synthesized compounds. In addition to the experimental results, the influence of solvent on the stability of compounds was investigated using DFT calculations at the B3LYP/6-311++G(d,p) level. Compared with solvent-free conditions, the stability of compounds was amplified in the presence of solvents and increased in the order of H_2_O > DMF > CH_3_CN > EtOH > THF. This trend was also in agreement with the experimental results. Theoretical data confirmed that the reaction performed best in water medium. Moreover, some electronic properties of these compounds, such as band gap, first ionization energy, electron affinity, electronic chemical potential, electrophilicity index, hardness and softness, were theoretically estimated in the presence of various solvents.

## Abstract 2

A combined experimental and theoretical investigation on the synthesis of polysubstituted quinolines in the presence of VB_1_ in aqueous media.

## Graphical abstract

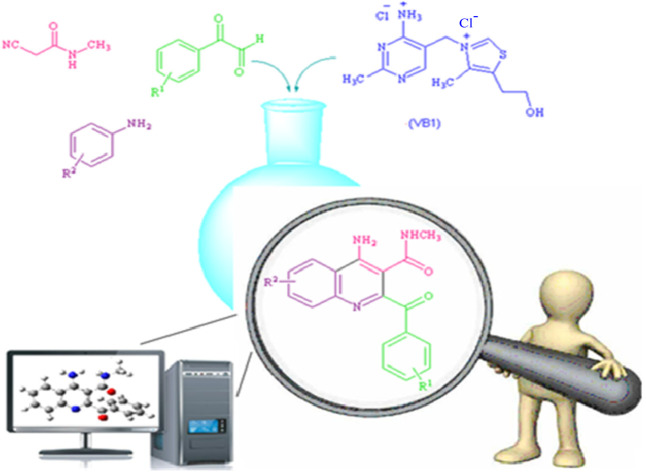

## 1. Introduction

The ability of multi-component reactions (MCRs) to concurrently integrate numerous reactants into a single reaction step has garnered significant interest in the field of chemical synthesis. In contrast to conventional organic reactions, which often include two or three reactants, MCRs enable the convergence of three or more components, leading to the efficient creation of complex molecular structures.^1–4^ From a biological standpoint, MCRs have a number of benefits that make them desirable tools for drug development.^5^

Nitrogen-containing heterocyclic compounds are essential in organic chemistry, playing crucial roles in biological synthesis and drug development.^6^ These compounds are found in nature and have diverse pharmacological activities, including anticancer, anti-HIV, antimalarial, and anti-tubercular properties.^7^ Their unique structure and diverse properties make them valuable in the fields of medicine, pharmaceuticals, materials science, and agriculture, urging their further exploration.^8–10^

**Fig. 1:**
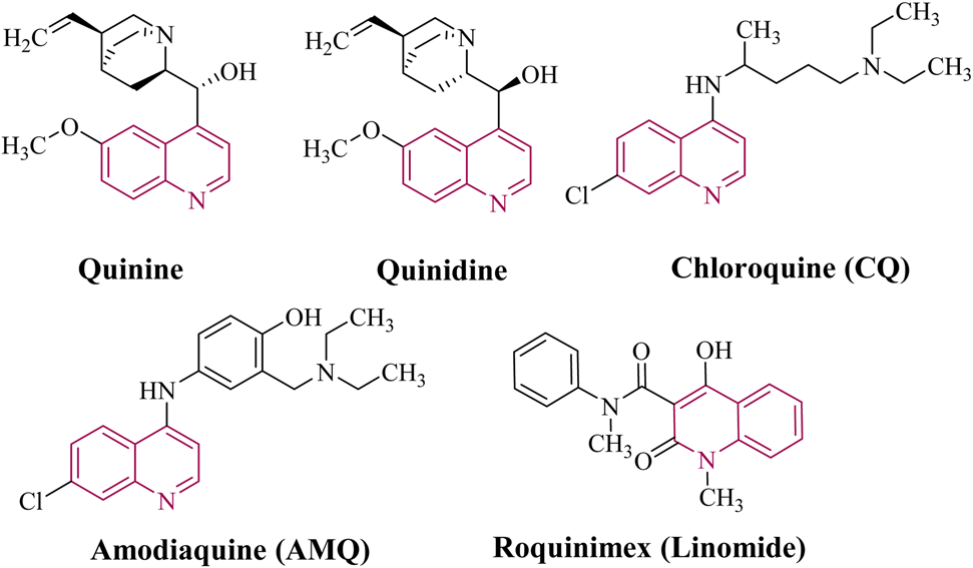
Natural and biologically active compounds containing a quinoline moiety.

Quinoline, an N-heterocyclic compound with a nitrogen-containing ring structure, was first isolated by Friedlieb Ferdinand Runge in 1834. Its unique structure and versatile applications have fascinated chemists for centuries.^11,12^ As a versatile organic synthesis building block with a unique benzene-pyridine ring structure, quinoline is a crucial precursor for the synthesis of various compounds owing to its wide reactivity and functionality.^13^ Quinoline's versatility facilitates diverse transformations, introducing functional groups at different positions and enabling the creation of novel compounds with desired features through nucleophilic and electrophilic substitution processes.^14^ Quinoline's structure is crucial for its biological activities,^15,16^ and naturally occurring alkaloids, such as quinine and quinidine, contain a quinoline moiety, which has been used for medicinal applications for centuries (Fig. 1).^17^ Quinoline derivatives have shown promise in drug development owing to their ability to modify their structure, leading to improved drug candidates. They have been used in treating various diseases and in the synthesis of dyes, agrochemicals, and advanced materials, such as conducting polymers and luminescent compounds.^18^

4-Aminoquinoline is a form of quinoline with an amino group at the 4-position of the quinoline. The compound has been used as a precursor for the synthesis of its derivatives.^19^ Most frequently used medications for treating malaria have a 4-aminoquinoline scaffold.^20^ The well-known antimalarial medication chloroquine (CQ) has a 4-aminoquinoline scaffold (Fig. 1). As an antimalarial, it works against the asexual form of the malaria parasite in the stage of its life cycle within the red blood cell.^21^ The structure related to 4-aminoquinoline, chloroquine, was identified in 1934.^22^ This drug is on the list of essential drugs of the World Health Organization and is available as a generic drug.^23^ Additionally, CQ has been shown to have antiviral properties against human HIV-1 (ref. 24 and 25) and the agents that cause severe acute respiratory syndrome (SARS).^26^ Amodiaquine (AMQ) can be mentioned among other compounds of 4-aminoquinoline (Fig. 1). This compound is used in the treatment of malaria, including *Plasmodium falciparum* malaria resistant to chloroquine.^27,28^ AMQ was first synthesized in 1948.^29^ This medicine is on the list of essential medicines of the World Health Organization. AMQ has become an important drug in combination therapy for the treatment of malaria in Africa. Moreover, 4-aminoquinoline derivatives are used as anti-asthmatic, anti-bacterial, anti-fungal, and anti-inflammatory agents.^19^ 4-Aminoquinoline compounds can be synthesized in various important ways including the condensation of appropriate amines with substituted quinolines, fusion of 4,7-dichloroquinoline with an amino group of side chains, methylation or formylation routes for *N*-methyl derivatives, butoxide-mediated synthesis from alkyl nitriles and aminobenzaldehydes, and imidoylative Sonogashira coupling followed by cyclization.^30–32^ In summary, the development of new 4-aminoquinoline compounds with high antimalarial potency and low toxicity is an ongoing area of research.

Quinoline-3-carboxamide is an important heterocyclic scaffold extensively studied in medicinal chemistry. It consists of a pyridine ring system fused with a benzene ring, substituted with a carboxamide group at the 3-position. This scaffold allows for various structural modifications and substitutions, leading to a diverse range of quinoline-3-carboxamide derivatives with potential biological activities. They have been shown to have a wide range of applications including as antimicrobial, antiviral, and anticancer agents.^33^ Quinoline-3-carboxamide compounds such as roquinimex (Linomide) (Fig. 1) exhibit immunomodulatory effects, including anti-inflammatory and anti-allergic properties, enhancing cell-mediated immunity and improving tumor surveillance.^34^ Quinoline-3-carboxamide compounds have been reported as potential inhibitors of ATM kinase and key mediators of the DNA damage response (DDR), which make these compounds valuable in cancer treatment.^35^ Quinoline-3-carboxamide derivatives have been shown to activate natural killer (NK) cells *via* the aryl hydrocarbon receptor, which increases their cytotoxicity against tumor cells and augments their immunoregulatory effects on dendritic cells.^36^ The synthesis of quinoline-3-carboxamide derivatives can be achieved through various methods including the Doebner–von Miller, Skraup, Vilsmeier–Haack, Combes, Friedlander, and Knorr synthesis, as well as copper-catalyzed reactions and cyclization reactions involving different starting materials.^23^ In summary, research on compounds with this scaffold is a promising approach for developing new chemotherapeutic agents.

Therefore, to broaden the scope of quinoline derivatives, we decided to design a series of new polysubstituted quinolines, where benzoyl, carboxamide, and amino groups were introduced at the positions of two, three, and four from the quinoline moiety, respectively. The introduction of different functional groups on quinoline moiety, especially carboxamide and amino groups, may further alter the properties of quinoline derivatives for pharmacological and biological purposes.

Thiamin hydrochloride (VB_1_) has been employed as an ecofriendly, cheap, nontoxic, easily accessible, and remarkable catalyst for the one-pot multi-component synthesis of various heterocyclic compounds.^37–40^ VB_1_ has also been used as a catalyst in organic transformations such as Knoevenagel condensation, Michael addition and cyclization.^41,42^

In continuation of our previous works to establish the one-pot multi-component strategies for the synthesis of the new heterocycles with potential biological activities,^43,44^ herein, we report the synthesis of 4-amino-2-benzoylquinoline-3-carboxamide derivatives by the reaction of 2-cyano-*N*-methylacetamide, arylglyoxals, and arylamines in the presence of VB_1_. Moreover, the influence of solvent on the stability and electronic properties of compounds was theoretically investigated using DFT calculations.

## 2. Results and discussion

**Scheme 1:**
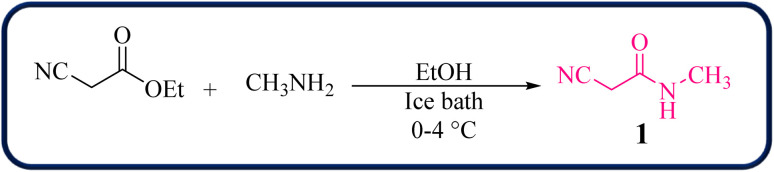
Synthesis of 2-cyano-*N*-methylacetamide.

To initiate our study, 2-cyano-*N*-methylacetamide 1 was achieved *via* the reaction of ethyl cyanoacetate and methyl amine in the presence of EtOH at 0–4 °C for 2 hours (Scheme 1). The 2-cyano-*N*-methylacetamide 1 compound was identified by comparison of its physical and spectral data with those of authentic samples.^45^

Then, the reaction of 2-cyano-*N*-methylacetamide 1 (1.0 mmol), 4-chlorophenylglyoxal (2a, 1.0 mmol), and 4-methoxyaniline (3a, 1.0 mmol) was performed under various reaction conditions for the synthesis of 4-amino-2-(4-chlorobenzoyl)-6-methoxy-*N*-methylquinoline-3-carboxamide 4a as a model reaction to establish the best reaction conditions (Table 1).

**Table 1:** Optimization of the reaction conditions for the synthesis of 4-amino-2-(4-chlorobenzoyl)-6-methoxy-*N*-methylquinoline-3-carboxamide 4a

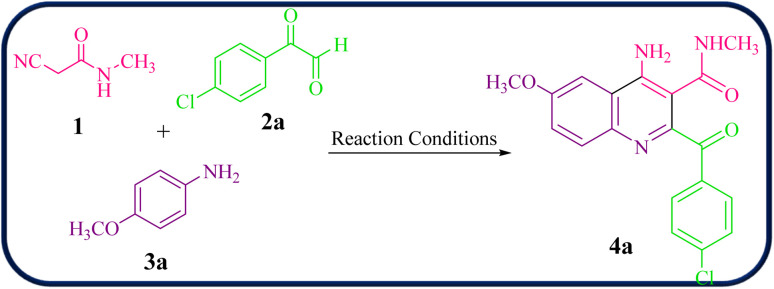				
Entry	Solvent	Catalyst (mol%)	Temp. a(°C)	Yield b(%)
1	—	VB_1_ (10)	r.t.	N.R.
2	DMF	VB_1_ (10)	r.t.	N.R.
3	EtOH	VB_1_ (10)	r.t.	N.R.
4	CH_3_CN	VB_1_ (10)	r.t.	N.R.
5	THF	VB_1_ (10)	r.t.	N.R.
6	H_2_O	VB_1_ (10)	r.t.	40
7	H_2_O	—	r.t.	N.R.
8	DMF	VB_1_ (10)	Reflux	Trace
9	EtOH	VB_1_ (10)	Reflux	Trace
10	CH_3_CN	VB_1_ (10)	Reflux	Trace
11	THF	VB_1_ (10)	Reflux	Trace
12	H_2_O	VB1 (10)	Reflux^c^	65
13	**H**_**2**_ **O**	**VB**_**1**_ **(15)**	**Reflux^c^**	**75**
14	H_2_O	VB_1_ (20)	Reflux^c^	75
15	H_2_O	TBAB (15)	Reflux	55
16	H_2_O	DMAP·HCl (15)	Reflux	50
17	H_2_O	AlCl_3_ (15)	Reflux	30
18	H_2_O	CuCl_2_ (15)	Reflux	35
19	H_2_O	ZnO (15)	Reflux	30

a Reaction conditions: solvent = 5 mL; reaction time = 12 h.

b Isolated yield.

c Reaction time = 6 h.

To obtain optimal reaction conditions, several factors including solvent, catalyst, and temperature were investigated, the results are presented in Table 1. The reaction was investigated in different solvents such as H_2_O, EtOH, CH_3_CN, THF, and DMF, and under solvent-free conditions at room temperature in the presence of 10 mol% VB_1_. No reaction occurred under solvent-free conditions and in the other solvents except H_2_O, and it was found that H_2_O is the best solvent for this reaction (Table 1, entry 6, yield 40%). Moreover, the desired reaction was performed without a catalyst in H_2_O at room temperature. It was found that 4a was not obtained after 12 h (Table 1, entry 7). Then, we observed that the reaction temperature also has an important influence on the reaction. Therefore, the reaction was carried out at room temperature in H_2_O for 12 h, the product was formed in 40% yield, but under reflux conditions for 6 h, the product was formed in 65% yield (Table 1, entries 6 and 12). Of course, in other solvents, in the presence of VB_1_ as the catalyst, the yield of the product was negligible under reflux conditions (Table 1, entries 8–11).

In addition to VB_1_ as the catalyst, other quaternary ammonium halides such as tetrabutylammonium bromide (TBAB) and *N*,*N*-dimethylaminopyridine hydrochloride (DMAP·HCl) were also tested under similar reaction conditions (Table 1, entries 15 and 16), but only 50–55% of product yields were obtained. Moreover, the reaction in the presence of catalysts including AlCl_3_, CuCl_2_ and ZnO under similar reaction conditions (Table 1, entries 17–19) did not achieve good yields.

Then, the same reaction was carried out in the presence of catalysts including AlCl_3_, CuCl_2_ and ZnO under similar reaction conditions (Table 1, entries 15–17), but the product yields were only 30–35%. Finally, we also observed that the mol% of VB_1_ as a catalyst could have an important influence on the reaction (Table 1, entries 12–14). When a larger amount of VB1 (15 mol%) was tested, a higher yield of 75% was obtained in H_2_O under reflux conditions (Table 1, entry 13). Notably, no change was detected in the yield after adding more amounts of VB_1_ (20 mol%) in H_2_O under reflux conditions (Table 1, entry 14). Thus, the optimized reaction conditions to prepare 4a was the use of 2-cyano-*N*-methylacetamide 1 (1.0 mmol), 4-chlorophenylglyoxal (2a, 1.0 mmol), and 4-methoxyaniline (3a, 1.0 mmol) in the presence of VB_1_ (15 mol%) in H_2_O under reflux conditions for 6 h (Table 1, entry 13).

Encouraged by these results, we further employed different arylglyoxals and arylamines with 2-cyano-*N*-methylacetamide to confirm the universality of this procedure under the optimized reaction condition (Table 2). In all cases tested, the reaction went smoothly, giving desired products in good yields. As can be seen from Table 2, the electronic effects and the nature of substituents on arylglyoxal 2 and arylamine 3 resulted in products with different reaction yields. Various substrates 2 and 3 with different substituents R^1^ and R^2^ on the aromatic rings were examined. To our delight, both electron-rich and electron-deficient groups (R^1^ and R^2^) in substrates 2 and 3 successfully afforded the desired products in good yields. Among them, if both substituents R^1^ and R^2^, or one of the substituents on arylglyoxals and arylamines, are electron-withdrawing groups, product 4a–i is synthesized with a relatively high yield. Moreover, the steric hindrance of substituents on substrates has no significant effect on the rate of the reactions.

**Table 2:** Synthesis of 4-amino-2-benzoyl-*N*-methylquinoline-3-carboxamide derivatives 4a–i

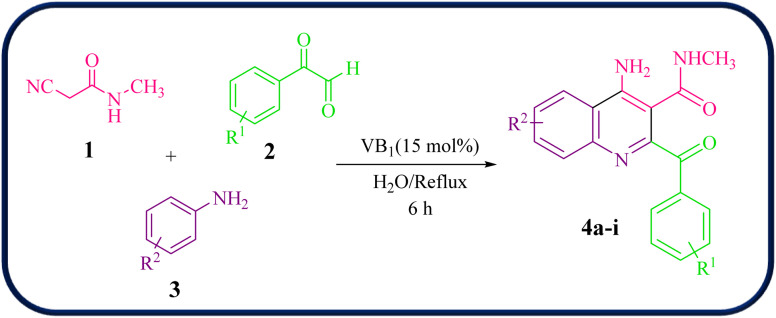				
Compound	R^1^	R^2^	Product	Yield a(%)
4a	4-Cl	4-OCH_3_	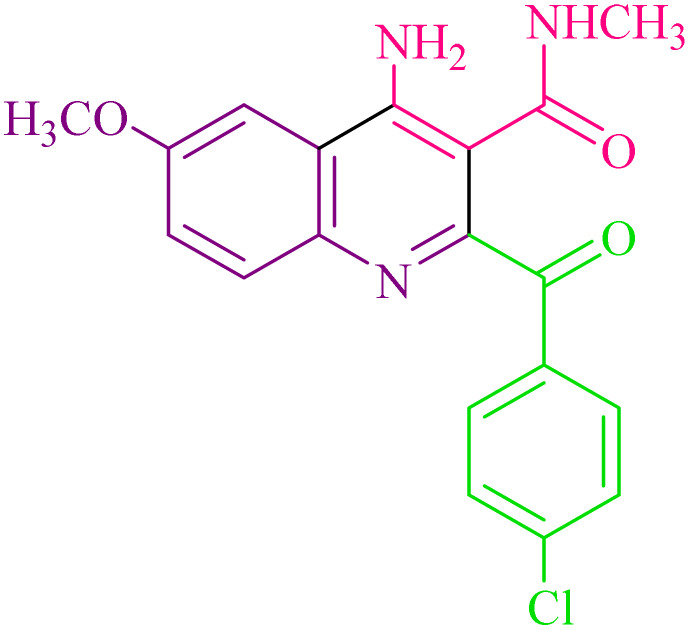	75
4b	4-Cl	4-CH_3_	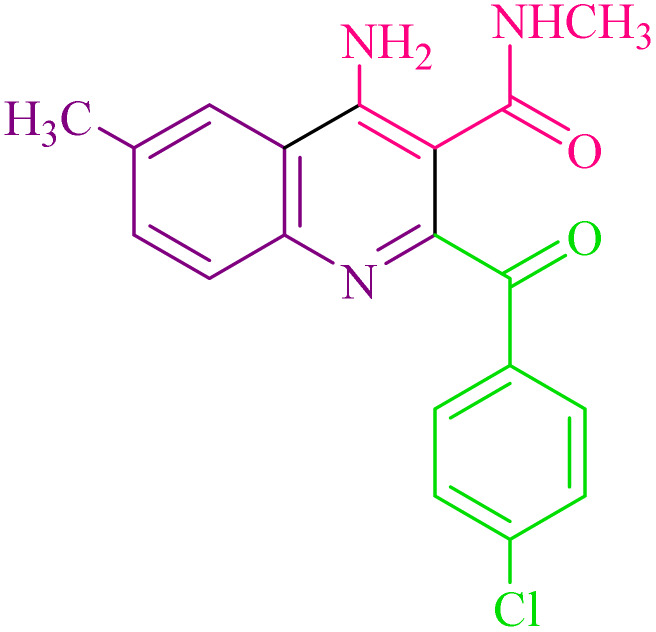	78
4c	4-Cl	4-Cl	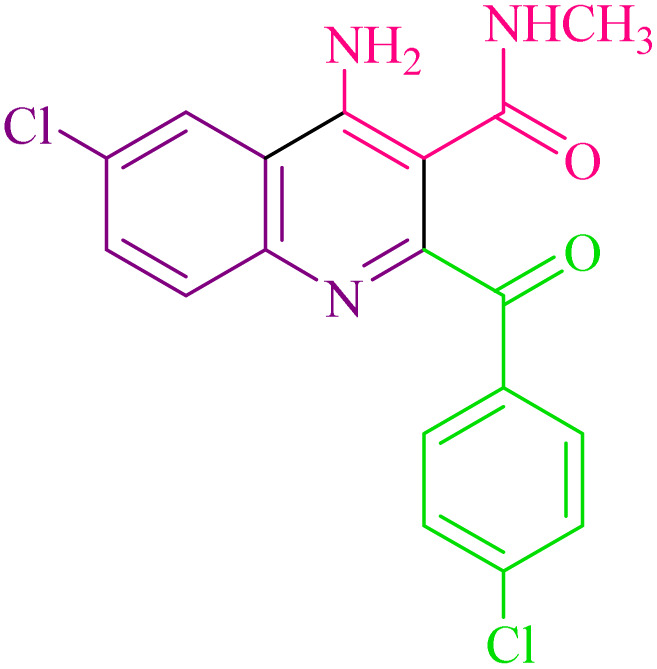	85
4d	4-H	4-Cl	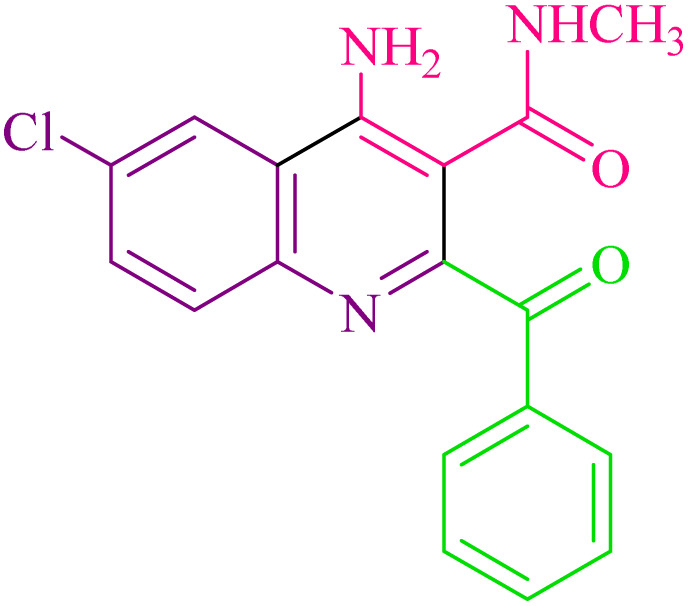	80
4e	4-Br	4-Cl	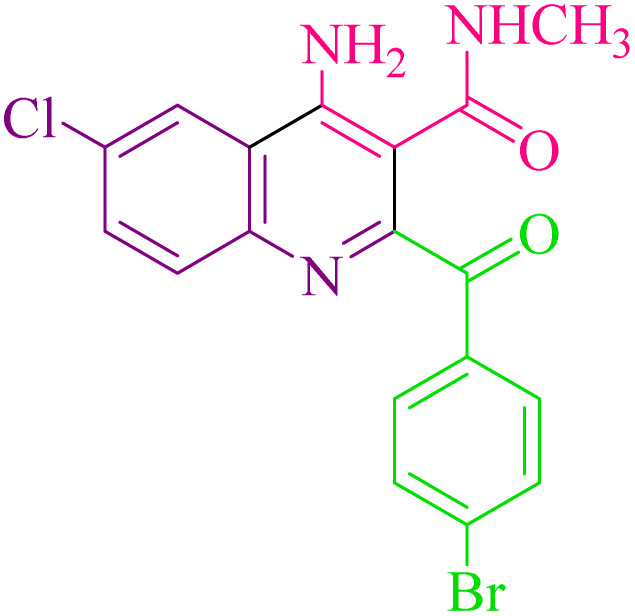	85
4f	4-Br	4-Br	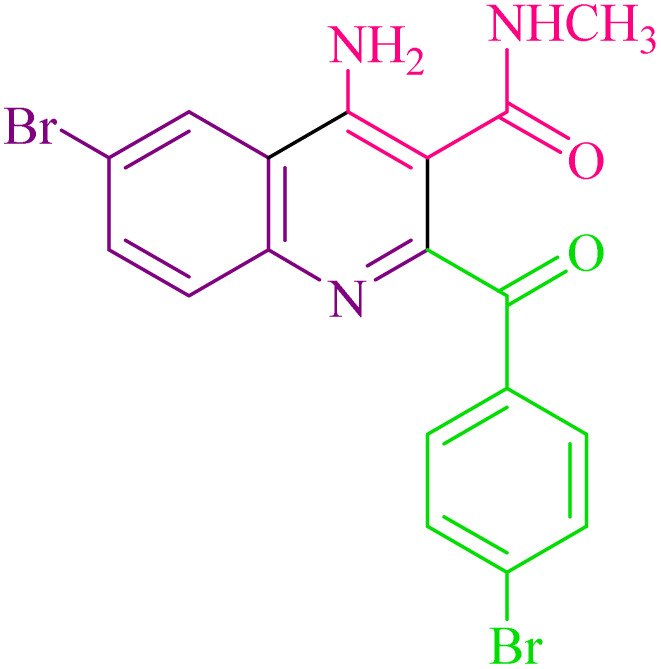	78
4g	4-CH_3_	2-Br	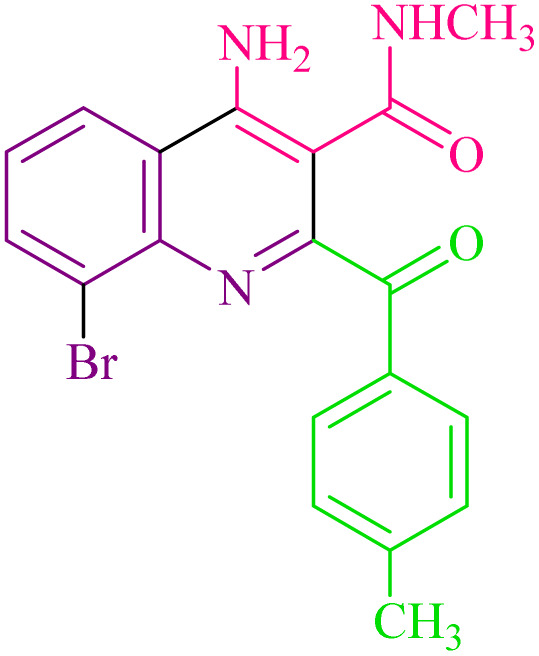	75
4h	4-OCH_3_	4-Cl	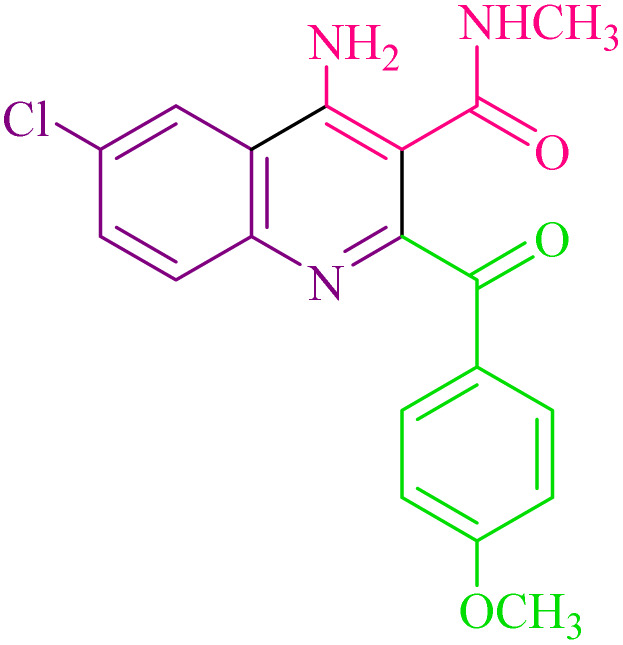	72
4i	4-CH_3_	4-CH_3_	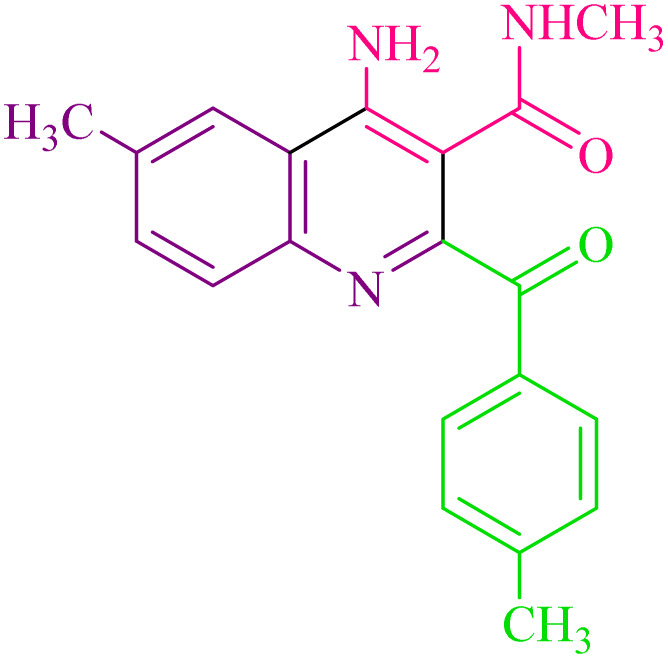	65

a Isolated yield.

**Fig. 2:**
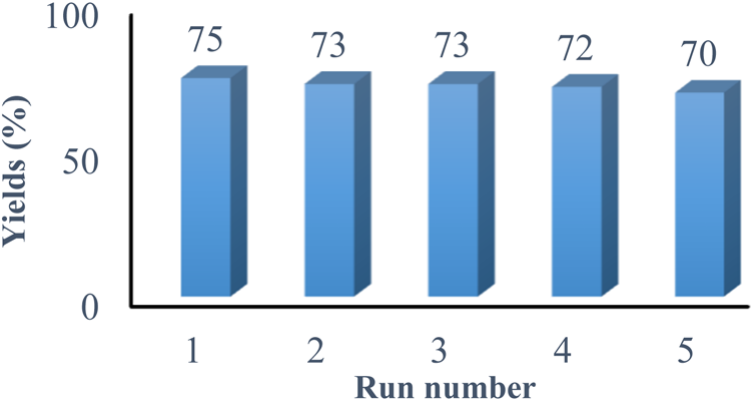
Reusability study of VB_1_ in the synthesis of 4a.

Moreover, the reusability of the catalyst was examined using the reaction of 2-cyano-*N*-methylacetamide 1, 4-chlorophenylglyoxal 2a, and 4-methoxyaniline 3a under the optimized reaction conditions. For this purpose, after completion of the reaction, the solvent was removed under reduced pressure. The reaction mixture was triturated with ethyl acetate and filtered. The catalyst was collected from the residue, washed with ethyl acetate, dried and used for the next cycle. We observed that the catalyst could be run for five times without any appreciable decrease in yield (Fig. 2).

To the best of our knowledge, all the synthesized compounds 4a–i are unknown and were characterized by IR, ^1^H and ^13^C-NMR and CHN analysis. For instance, the IR spectrum of 4-amino-2-(4-chlorobenzoyl)-6-methoxy-*N*-methylquinoline-3-carboxamide 4a showed bands at 3452 cm^−1^ and 3257 cm^−1^ for NH_2_, 3183 cm^−1^ for NH, 1704 cm^−1^ and 1684 cm^−1^ for C=O, and 1617 cm^−1^ and 1512 cm^−1^ for C=C aromatic groups. In the ^1^H NMR spectrum of compound 4a, a doublet signal at *δ* = 2.75 ppm with a coupling constant of 5.82 Hz for the methyl, a singlet signal at *δ* = 3.72 ppm for the methoxy group, a doublet signal at *δ* = 7.06 ppm with a coupling constant of 5.82 Hz for NH proton, and a singlet signal, which was integrated, for two protons at *δ* = 7.34 ppm for the NH_2_ protons were observed. Moreover, the aromatic protons resonated in the region *δ* = 7.68-8.20 ppm. The ^13^C NMR spectrum of compound 4a showed 17 distinct signals in agreement with the proposed structure.

**Scheme 2:**
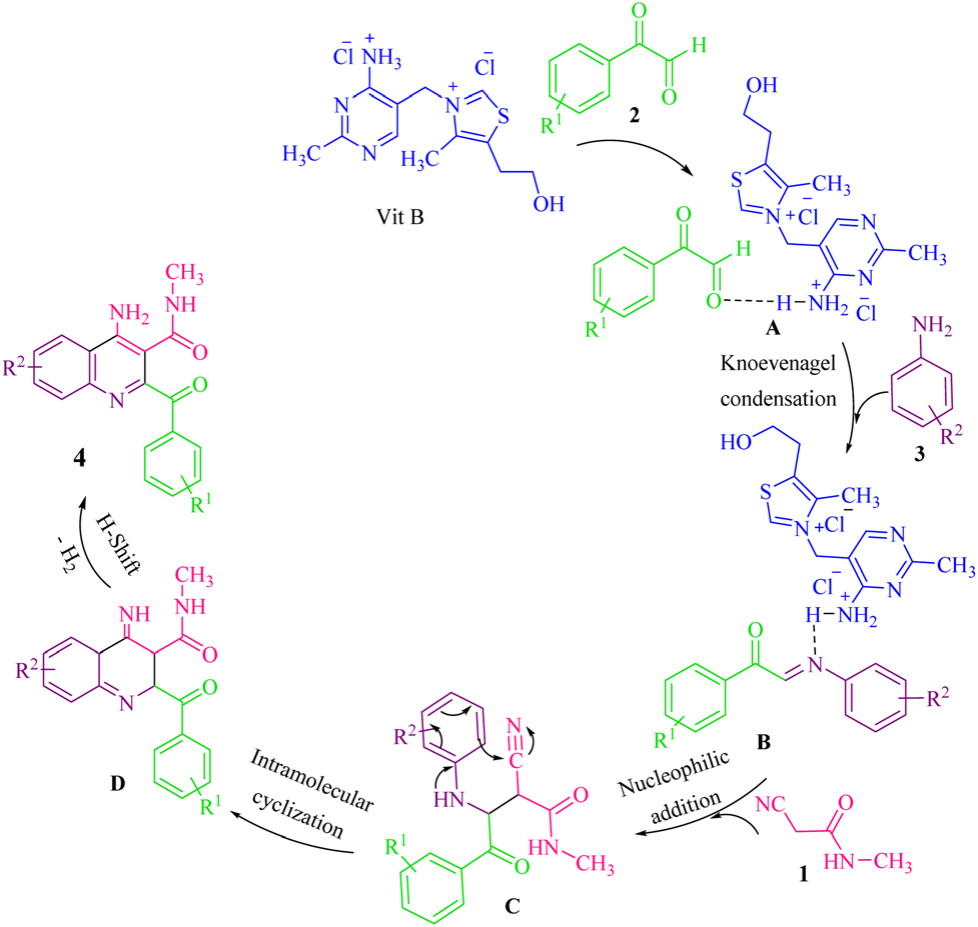
Proposed mechanism for the preparation of 4.

We propose a plausible mechanism for the one-pot reaction between arylglyoxal, arylamine, and 2-cyano-*N*-methylacetamide in the presence of VB_1_ (Scheme 2). Initially, the catalyst VB_1_ activates arylglyoxal 2 through the NH proton (intermediate A), so that the electrophilic nature of the carbonyl group increases, thereby facilitating the nucleophilic attack by arylamine 3 through the Knoevenagel condensation and forming intermediate B. The intermediate B, an iminone, which is further activated by the catalyst undergoes a nucleophilic attack by 2-cyano-*N*-methylacetamide 1 to obtain intermediate C. The intermediate C by an intramolecular cyclization reaction affords intermediate D, which is followed by oxidation and hydrogen shift to obtain the target molecule 4.

In order to investigate the stability of compounds in the presence of chosen solvents, the formation energy (Δ*E*) was theoretically obtained according to the following formula:Δ*E* = *E*_compound_ − Σ*E*_mon_where *E*_compound_ and *E*_mon_ are the optimized energies of compounds and each individual component monomer, respectively. Δ*E*_ZPE_, Δ*E*_Thermal_, Δ*H* and Δ*G* values were calculated similar to Δ*E*. As evident from the results in Tables S1–S5,† the negative energy values show an upward trend and the stability of compounds, in comparison with solvent-free conditions, is amplified in the presence of solvents, and increases in the order of H_2_O > DMF > CH_3_CN > EtOH > THF. This trend is in agreement with the experimental results. Theoretical data confirm that the reaction performs best in water.

Subsequently, the influence of solvents on the electronic properties of compounds was also investigated. As given in Tables S6–S11,† the band gap (*E*_g_), first ionization energy (IE), electron affinity (EA), electronic chemical potential (*μ*), electrophilicity index (*ω*), hardness (*η*) and softness (*σ*) were estimated in the presence of solvents.

The conductivity of compounds can be evaluated using the energy band gap as follows:*E*_g_ = *E*_LUMO_ − *E*_HOMO_Here, *E*_HOMO_ and *E*_LUMO_, respectively, correspond to the energies of the highest occupied molecular orbital (HOMO) and the lowest unoccupied molecular orbital (LUMO). Since conductivity is inversely proportional to the energy band gap, the conductivity of compounds may be amplified in the order of H_2_O > DMF > CH_3_CN > EtOH > THF.

The first ionization energy (IE) and electron affinity (EA) were also estimated using Koopmans' theorem^46^ according to IE = –*E*_HOMO_ and EA = –*E*_LUMO_. As observed from Tables S6–S11,† the IE values of compounds increase in the following order: THF > EtOH > CH_3_CN > DMF > H_2_O. An opposite trend was found for EA values.

The electronic chemical potential is usually used to examine the escaping tendency of the electron in the system. The electronic chemical potential (*μ*) and the electronegativity (*χ*) can be measured by electron affinity and first ionization energy as follows:^47^
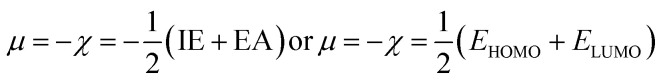


One can see from Tables S6–S11† that the absolute value of *μ* in solvents is enhanced in the order of THF > EtOH > CH_3_CN > DMF > H_2_O.

The ability of the compound to respond to an electric field and acquire an electric dipole moment depends on its polarizability. The global chemical hardness (*η*) and chemical softness (*σ*) can be used to measure the polarizability. The definitions of these quantities are as follows:^48^
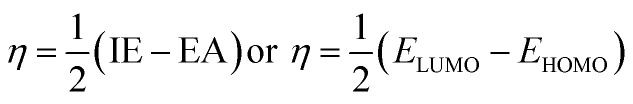
and
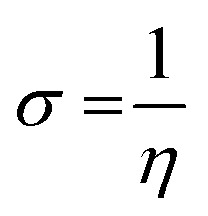


A small band gap automatically means small excitation energies to the manifold of excited states. Thus, soft compounds, with a small band gap, will be more polarizable than hard cases.^49^ The global chemical hardness of compounds in solvents is amplified in the order of THF > EtOH > CH_3_CN > DMF > H_2_O.

The concept of electrophilicity index (*ω*), proposed by Parr *et al.*,^50^ is a measure of the propensity of electron acceptors to acquire the maximal number of electrons from the environment. It can be calculated using *μ* and *η* parameters as follows:
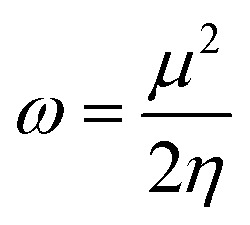


As can be seen in Tables S6–S11,† the *ω* values in solvents increase in the order of H_2_O > DMF > CH_3_CN > EtOH > THF.

## 3. Experimental

### 3.1. General

All chemicals of high-grade quality were purchased from Aldrich and Merck and used without further purification. All products were obtained by reaction at reflux in water as the solvent. The reactions were monitored by TLC and all yields refer to isolated products. All melting points were obtained using a Barnstead Electro thermal 9200 apparatus and were uncorrected. IR spectra were recorded using a Bruker FT-IR Equinax-55 spectrophotometer in KBr with absorption in cm^−1^. NMR spectra were recorded using a Varian model UNITY Inova 500 MHz spectrometer (^1^H: 500 MHz; ^13^C: 125 MHz) in DMSO-*d*_6_ using TMS as an internal standard. Elemental analysis was performed using a Carlo Erba EA 1108 instrument.

### 3.2. Synthetic procedures

#### 3.2.1. General experimental procedure for the synthesis of compounds 4a–i

A mixture of 2-cyano-*N*-methylacetamide 1 (1.0 mmol), arylglyoxal 2 (1.0 mmol), and arylamine 3 (1.0 mmol) in the presence of 15 mol% of catalyst VB_1_ was stirred in H_2_O (5 mL) under reflux conditions for 6 hours. After the completion of the reaction monitored by TLC, the solvent was removed under reduced pressure. The reaction mixture was triturated with ethyl acetate and filtered. The catalyst was collected from the residue and washed with ethyl acetate, dried and used for the next cycle. The crude product was purified by plate chromatography (20 × 20 cm) using *n*-hexane/EtOAc (1 : 1) as an eluent to obtain pure compounds 4a–i (65-85%).

##### 3.2.1.1. 4-Amino-2-(4-chlorobenzoyl)-6-methoxy-*N*-methylquinoline-3-carboxamide (4a)

Yellow oil; FT-IR (KBr, cm^−1^): 3452 and 3257 (NH_2_), 3183 (NH), 1704 and 1684 (C=O), 1617 and 1512 (C=C aromatic groups); ^1^H NMR (500 MHz, DMSO-*d*_6_) *δ*: 2.75 (d, *J* = 5.82 Hz, 3H, CH_3_), 3.72 (s, 3H, OCH_3_), 7.06 (d, *J* = 5.82 Hz, 1H, NH), 7.34 (s, 2H, NH_2_), 7.62 (d, *J* = 8.40 Hz, 1H, ArH), 7.68 (dd, ^1^*J* = 6.85, ^2^*J* = 3.55 Hz, 2H, ArH), 7.74 (dd, ^1^*J* = 7.80, ^2^*J* = 4.1 Hz, 2H, ArH), 7.76 (d, *J* = 8.40 Hz, 1H, ArH), 8.20 (d, *J* = 2.70 Hz, 1H, ArH) ppm; ^13^C NMR (125 MHz, DMSO-*d*_6_) *δ*: 27.18, 71.11, 113.91, 114.75, 120.51, 125.33, 126.33, 128.65, 129.44, 131.54, 131.66, 133.32, 147.04, 158.99, 162.41, 166.89, 196.67 ppm. Anal. calcd for C_19_H_16_ClN_3_O_3_ (369.81): C, 61.71; H, 4.36; N, 11.36; found: C, 61.06; H, 4.12; N, 11.94%.

##### 3.2.1.2. 4-Amino-2-(4-chlorobenzoyl)-*N*,6-dimethylquinoline-3-carboxamide (4b)

Yellow oil; FT-IR (KBr, cm^−1^): 3451 and 3258 (NH_2_), 3185 (NH), 1707 and 1681 (C=O), 1617 and 1513 (C=C aromatic groups); ^1^H NMR (500 MHz, DMSO-*d*_6_) *δ*: 2.33 (s, 3H, CH_3_), 2.72 (d, *J* = 4.70 Hz, 3H, CH_3_), 6.95 (d, *J* = 4.70 Hz, 1H, NH), 7.20 (s, 2H, NH_2_), 7.23 (s, 1H, ArH), 7.32 (d, *J* = 8.30 Hz, 2H, ArH), 7.35 (d, *J* = 8.90 Hz, 1H, ArH), 7.59 (d, *J* = 8.90 Hz, 1H, ArH), 7.64 (d, *J* = 8.40 Hz, 2H, ArH) ppm; ^13^C NMR (125 MHz, DMSO-*d*_6_) *δ*: 21.38, 29.40, 113.41, 115.54, 121.19, 124.32, 125.32, 129.19, 130.27, 131.56, 131.87, 135.28, 139.48, 149.33, 159.37, 163.12, 209.20 ppm. Anal. calcd for C_19_H_16_ClN_3_O_2_ (353.81): C, 64.50; H, 4.56; N, 11.88; found: C, 65.12; H, 4.87; N, 11.29%.

##### 3.2.1.3. 4-Amino-6-chloro-2-(4-chlorobenzoyl)-*N*-methylquinoline-3-carboxamide (4c)

Yellow oil; FT-IR (KBr, cm^−1^): 3452 and 3258 (NH_2_), 3185 (NH), 1706 and 1681 (C=O), 1616 and 1489 (C=C aromatic groups); ^1^H NMR (500 MHz, DMSO-*d*_*6*_) *δ*: 2.72 (d, *J* = 4.72 Hz, 3H, CH_3_), 7.03 (d, *J* = 4.72 Hz, 1H, NH), 7.31 (s, 2H, NH_2_), 7.35 (dd, ^1^*J* = 6.65, ^2^*J* = 2.25 Hz, 2H, ArH), 7.59 (d, *J* = 7.65 Hz, 1H, ArH), 7.60 (d, *J* = 7.85 Hz, 1H, ArH), 7.73 (dd, ^1^*J* = 6.55, ^2^*J* = 2.15 Hz, 2H, ArH), 8.26 (d, *J* = 1.95 Hz, 1H, ArH) ppm; ^13^C NMR (125 MHz, DMSO-*d*_6_) *δ*: 29.43, 115.19, 120.54, 122.47, 125.82, 126.00, 127.16, 130.07, 132.77, 138.42, 141.75, 143.26, 147.62, 159.59, 162.88, 192.01 ppm. Calcd for C_18_H_13_Cl_2_N_3_O_2_ (374.22): C, 57.77; H, 3.50; N, 11.23; found: C, 58.39; H, 3.73; N, 10.74%.

##### 3.2.1.4. 4-Amino-2-benzoyl-6-chloro-*N*-methylquinoline-3-carboxamide (4d)

Yellow oil; FT-IR (KBr, cm^−1^): 3448 and 3253 (NH_2_), 3181 (NH), 1704 and 1679 (C=O), 1605 and 1492 (C=C aromatic groups); ^1^H NMR (500 MHz, DMSO-*d*_*6*_) *δ*: 2.73 (d, *J* = 4.67 Hz, 3H, CH_3_), 6.99 (d, *J* = 4.67 Hz, 1H, NH), 7.25 (s, 2H, NH_2_), 7.34 (d, *J* = 7.95 Hz, 2H, ArH), 7.41 (t, *J* = 8.90 Hz, 2H, ArH), 7.51 (t, *J* = 6.50 Hz, 1H, ArH), 7.59 (d, *J* = 8.80 Hz, 1H, 16ArH), 7.75 (d, *J* = 8.80 Hz, 1H, ArH), 8.26 (d, *J* = 1.90 Hz, 1H, ArH) ppm; ^13^C NMR (125 MHz, DMSO-*d*_6_) *δ*: 25.92, 115.10, 123.25, 123.79, 124.82, 125.21, 126.28, 129.82, 138.99, 144.20, 148.77, 156.10, 158.81, 159.67, 162.62, 192.64 ppm. Calcd for C_18_H_14_ClN_3_O_2_ (339.78): C, 63.63; H, 4.15; N, 12.37; found: C, 64.27; H, 4.31; N, 12.03%.

##### 3.2.1.5. 4-Amino-2-(4-bromobenzoyl)-6-chloro-*N*-methylquinoline-3-carboxamide (4e)

Yellow oil; FT-IR (KBr, cm^−1^): 3453 and 3259 (NH_2_), 3187 (NH), 1705 and 1682 (C=O), 1608 and 1493 (C=C aromatic groups); ^1^H NMR (500 MHz, DMSO-*d*_*6*_) *δ*: 2.72 (d, *J* = 4.40 Hz, 3H, CH_3_), 7.02 (d, *J* = 4.40 Hz, 1H, NH), 7.31 (s, 2H, NH_2_), 7.35 (dd, ^1^*J* = 8.90, ^2^*J* = 2.30 Hz, 2H, ArH), 7.59 (dd, ^1^*J* = 6.70, ^2^*J* = 2.17 Hz, 2H, ArH), 7.66 (d, *J* = 8.85 Hz, 1H, ArH), 7.73 (d, *J* = 8.85 Hz, 1H, ArH), 8.26 (d, *J* = 1.95 Hz, 1H, ArH) ppm; ^13^C NMR (125 MHz, DMSO-*d*_6_) *δ*: 29.40, 115.43, 119.49, 121.20, 126.02, 127.61, 129.19, 129.67, 132.78, 137.60, 144.80, 156.65, 157.97, 158.41, 160.13, 194.90 ppm. Calcd for C_18_H_13_BrClN_3_O_2_ (418.68): C, 51.64; H, 3.13; N, 10.04; found: C, 52.11; H, 3.19; N, 9.74%.

##### 3.2.1.6. 4-Amino-6-bromo-2-(4-bromobenzoyl)-*N*-methylquinoline-3-carboxamide (4f)

Yellow oil; FT-IR (KBr, cm^−1^): 3449 and 3256 (NH_2_), 3184 (NH), 1703 and 1679 (C=O), 1606 and 1490 (C=C aromatic groups); ^1^H NMR (500 MHz, DMSO-*d*_*6*_) *δ*: 2.72 (d, *J* = 4.65 Hz, 3H, CH_3_), 7.02 (d, *J* = 4.65 Hz, 1H, NH), 7.31 (s, 2H, NH_2_), 7.48 (dd, ^1^*J* = 6.70, ^2^*J* = 2.20 Hz, 2H, ArH), 7.54 (dd, ^1^*J* = 6.70, ^2^*J* = 2.17 Hz, 2H, ArH), 7.66 (d, *J* = 8.75 Hz, 1H, ArH), 7.73 (d, *J* = 8.70 Hz, 1H, ArH), 8.27 (d, *J* = 2.00 Hz, 1H, ArH) ppm; ^13^C NMR (125 MHz, DMSO-*d*_6_) *δ*: 29.41, 115.18, 119.84, 124.11, 126.01, 127.16, 132.10, 132.33, 132.56, 132.79, 138.01, 142.10, 150.47, 160.15, 162.91, 196.01 ppm. Calcd for C_18_H_13_Br_2_N_3_O_2_ (463.13): C, 46.68; H, 2.83; N, 9.07; found: C, 46.92; H, 2.88; N, 8.64%.

##### 3.2.1.7. 4-Amino-8-bromo-*N*-methyl-2-(4-methylbenzoyl)quinoline-3-carboxamide (4g)

Yellow oil; FT-IR (KBr, cm^−1^): 3453 and 3259 (NH_2_), 3186 (NH), 1704 and 1681 (C=O), 1614 and 1491 (C=C aromatic groups); ^1^H NMR (500 MHz, DMSO-*d*_*6*_) *δ*: 2.33 (s, 3H, CH_3_), 2.73 (d, *J* = 4.97 Hz, 3H, CH_3_), 6.92 (d, *J* = 4.97 Hz, 1H, NH), 7.17 (s, 2H, NH_2_), 7.21 (d, *J* = 7.35 Hz, 1H, ArH), 7.23 (d, *J* = 8.30 Hz, 1H, ArH), 7.32 (d, *J* = 8.30 Hz, 2H, ArH), 7.38 (t, *J* = 7.65 Hz, 1H, ArH), 7.65 (d, *J* = 8.30 Hz, 2H, ArH) ppm; ^13^C NMR (125 MHz, DMSO-*d*_6_) *δ*: 21.37, 29.42, 115.55, 120.52, 124.32, 125.33, 128.61, 130.26, 136.71, 139.48, 142.40, 149.34, 153.46, 155.04, 159.35, 163.11, 197.11 ppm. Calcd for C_19_H_16_BrN_3_O_2_ (398.26): C, 57.30; H, 4.05; N, 10.55; found: C, 56.71; H, 3.82; N, 10.69%.

##### 3.2.1.8. 4-Amino-6-chloro-2-(4-methoxybenzoyl)-*N*-methylquinoline-3-carboxamide (4h)

Yellow oil; FT-IR (KBr, cm^−1^): 3453 and 3258 (NH_2_), 3185 (NH), 1706 and 1681 (C=O), 1616 and 1494 (C=C aromatic groups); ^1^H NMR (500 MHz, DMSO-*d*_*6*_) *δ*: 2.72 (d, *J* = 4.65 Hz, 3H, CH_3_), 3.79 (s, 3H, OCH_3_), 6.91 (d, *J* = 4.65 Hz, 1H, NH), 6.94 (d, *J* = 8.70 Hz, 1H, ArH), 7.09 (dd, ^1^*J* = 6.90, ^2^*J* = 2.15 Hz, 2H, ArH), 7.14 (s, 2H, NH_2_), 7.18 (s, 1H, ArH), 7.30 (d, *J* = 8.75 Hz, 1H, ArH), 7.69 (dd, ^1^*J* = 6.85, ^2^*J* = 2.07 Hz, 2H, ArH) ppm; ^13^C NMR (125 MHz, DMSO-*d*_6_) *δ*: 29.43, 55.85, 114.04, 115.28, 120.66, 126.15, 129.08, 131.64, 131.74, 132.90, 138.84, 145.32, 149.61, 159.14, 160.39, 163.18, 193.91 ppm. Calcd for C_19_H_16_ClN_3_O_3_ (369.81): C, 61.71; H, 4.36; N, 11.36; found: C, 61.27; H, 4.28; N, 11.52%.

##### 3.2.1.9. 4-Amino-*N*,6-dimethyl-2-(4-methylbenzoyl)quinoline-3-carboxamide (4i)

Yellow oil; FT-IR (KBr, cm^−1^): 3453 and 3259 (NH_2_), 3180 (NH), 1707 and 1682 (C=O), 1617 and 1514 (C=C aromatic groups); ^1^H NMR (500 MHz, DMSO-*d*_*6*_) *δ*: 2.23 (s, 3H, CH_3_), 2.33 (s, 3H, CH_3_), 2.73 (d, *J* = 4.5 Hz, 3H, CH_3_), 6.92 (s, 1H, NH), 7.09 (d, *J* = 8.6 Hz, 1H, ArH), 7.17 (s, 2H, NH_2_), 7.22 (d, *J* = 7.40 Hz, 1H, ArH), 7.32 (d, *J* = 8.65 Hz, 2H, ArH), 7.44 (d, *J* = 8.55 Hz, 1H, ArH), 7.65 (d, *J* = 8.25 Hz, 2H, ArH) ppm; ^13^C NMR (125 MHz, DMSO-*d*_6_) *δ*: 21.30, 21.37, 29.47, 115.56, 119.58, 124.31, 125.32, 127.13, 129.24, 129.61, 130.25, 132.95, 136.20, 139.46, 149.32, 159.72, 163.13, 195.47 ppm. Calcd for C_20_H_19_N_3_O_2_ (333.39): C, 72.05; H, 5.74; N, 12.60; found: C, 71.59; H, 5.57; N, 12.36%.

### 3.3. Computational section

The energetic and geometrical properties have been optimized using Gaussian 09 suite of programs^51^ at the B3LYP/6-311++G(d,p) level. The compounds were investigated in gas phase and in water (H_2_O), acetonitrile (CH_3_CN), tetrahydrofuran (THF), ethanol (EtOH) and *N*,*N*-dimethylformamide (DMF) as solvents. For modeling the solvation effects, the polarizable continuum model (PCM) is usually used.^52–55^ The PCM creates a solute cavity using a set of overlapping spheres, so that meaningful data can be obtained.^56^ In this study, the influence of solvent on the stability and electronic properties of compounds was examined using the PCM during the optimization of structures of compounds at the B3LYP/6-311++G(d,p) level of theory. The frequency calculations were also used to estimate the thermodynamic functions of compounds such as zero-point energy (*E*_ZPE_), thermal energy (*E*_Thermal_), enthalpy (*H*) and Gibbs free energy (*G*).

## 4. Conclusions

In summary, we have designed an efficient protocol for the synthesis of 4-amino-2-benzoylquinoline-3-carboxamides *via* a one-pot three-component reaction of 2-cyano-*N*-methylacetamide, arylglyoxals, and arylamines using catalytic amounts of VB_1_. These new series of quinoline derivatives which contain a carboxamide group at the 3-position and an amino group at the 4-position are of great importance in medicinal chemistry. The use of readily available starting materials, simple reaction conditions, cheaply available and reusable catalysts, easy work-up procedure, and the high yield of products are the key features of this MCR protocol. Further studies and medicinal applications of these compounds are being investigated and will be reported in due course.

Moreover, the influence of solvent on the stability of compounds was theoretically investigated. As compared to solvent-free conditions, the stability of compounds is amplified in the presence of solvents, and increases in the order of H_2_O > DMF > CH_3_CN > EtOH > THF. This trend is in agreement with experimental results. Theoretical data confirm that the reaction performs best in water. Moreover, some electronic properties of these compounds, such as band gap, first ionization energy, electron affinity, electronic chemical potential, electrophilicity index, hardness and softness, were theoretically estimated in the presence of solvents.

## Data availability

The data supporting this article have been included as part of the ESI.†

## Author contributions

Mina Hajipour: writing – original draft, methodology, formal analysis, data curation. Hossein Mehrabi: writing – review & editing, validation, supervision, project administration, conceptualization. Hamid Reza Masoodi: writing – review, software, resources, investigation, data curation.

## Conflicts of interest

There are no conflicts to declare.

## Supplementary Material

RA-015-D5RA01461A-s001

RA-015-D5RA01461A-s002
