# Supplementary material for: Thiamine hydrochloride (VB1) in aqueous media catalyzed the synthesis of polysubstituted quinolines via a one-pot strategy: a combined experimental and theoretical investigation
Source: RSC Adv. 2025 Apr 24;15(17):13127–36. doi: 10.1039/d5ra01461a (PMC12018799; doi:10.1039/d5ra01461a)
Supplement: RA-015-D5RA01461A-s001 [file RA-015-D5RA01461A-s001.pdf]

Supplementary Information (SI) for RSC Advances.
This journal is © The Royal Society of Chemistry 2025

Thiamine hydrochloride (VB_1) in aqueous medium catalyzed synthesis of polysubstituted quinolines *via* one-pot strategy: A combined experimental and theoretical investigation

Mina Hajipour, Hossein Mehrabi*, Hamid Reza Masoodi

Department of Chemistry, Vali-e-Asr University of Rafsanjan, Rafsanjan, Iran

**Corresponding author. E-mail: h.mehrabi@vru.ac.ir*

2. Experimental Section

2.1. General

All chemicals were purchased from Aldrich and Merck with high-grade quality and used without any purification. All products were obtained by reaction at reflux in water as solvent. The reactions were monitored by TLC and all yields refer to isolated products. All melting points were obtained by Barnstead Electro thermal 9200 apparatus and are uncorrected. IR spectra were recorded on a Bruker FT-IR Equinax-55 spectrophotometer in KBr with absorption in cm^{-1}. The NMR spectra were recorded on a Varian model UNITY Inova 500 MHz spectrometer (^{1}H: 500 MHz, ^{13}C: 125 MHz) in DMSO-d_6 using TMS as an internal standard. Elemental analysis were performed using a Carlo Erba EA 1108 instrument.

2.2. Synthetic procedures

*2.2.1. General experimental procedure for the synthesis of compounds **4a-i**.*

A mixture of 2-cyano-*N*-methylacetamide **1** (1.0 mmol), arylglyoxal **2** (1.0 mmol), and arylamine **3** (1.0 mmol) in the presence of 15 mol% of catalyst VB_1 was stirred in H_2O (5 mL) under reflux conditions for 6 hours. After the completion of the reaction monitored by TLC, the catalyst was isolated by simple filtration and the reaction mixture was extracted with ethyl acetate and. The product in ethyl acetate, and the solvent removed under reduced pressure and the crude product was purified by plate chromatography (20×20 cm) using n-hexane/EtOAc (1:1) as eluent to give the pure compounds **4a-i** (65-85%).

*2.2.1.1. 4-Amino-2-(4-chlorobenzoyl)-6-methoxy-N-methylquinoline-3-carboxamide **(4a)**.*

Yellow oil; FT-IR (KBr, cm^{-1}): 3452 and 3257 (NH_2), 3183 (NH), 1704 and 1684 (C═O), 1617 and 1512 (C═C aromatic groups); ^{1}H NMR (500 MHz, DMSO-d_6) δ: 2.75 (d, J = 5.82 Hz, 3H, CH_3), 3.72 (s, 3H, OCH_3), 7.06 (d, J = 5.82 Hz, 1H, NH), 7.34 (s, 2H, NH_2), 7.62 (d, J = 8.40 Hz, 1H, ArH), 7.68 (dd, ^{1}J = 6.85, ^{2}J = 3.55 Hz, 2H, ArH), 7.74 (dd, ^{1}J = 7.80, ^{2}J = 4.1 Hz, 2H, ArH), 7.76 (d, J = 8.40 Hz, 1H, ArH), 8.20 (d, J = 2.70 Hz, 1H, ArH) ppm; ^{13}C NMR (125 MHz, DMSO-d_6) δ: 27.18, 71.11, 113.91, 114.75, 120.51, 125.33, 126.33, 128.65, 129.44, 131.54, 131.66, 133.32, 147.04, 158.99, 162.41, 166.89, 196.67 ppm. Anal. Calcd for $C_{19}H_{16}ClN_3O_3$ (369.81): C, 61.71; H, 4.36; N, 11.36; Found: C, 61.06; H, 4.12; N, 11.94 %.

*2.2.1.2. 4-Amino-2-(4-chlorobenzoyl)-N,6-dimethylquinoline-3-carboxamide **(4b)**.*

Yellow oil; FT-IR (KBr, cm^{-1}): 3451 and 3258 (NH_2), 3185 (NH), 1707 and 1681 (C═O), 1617 and 1513 (C═C aromatic groups); ^{1}H NMR (500 MHz, DMSO-d_6) δ: 2.33 (s, 3H, CH_3), 2.72 (d, J = 4.70 Hz, 3H, CH_3), 6.95 (d, J = 4.70 Hz, 1H, NH), 7.20 (s, 2H, NH_2), 7.23 (s, 1H, ArH), 7.32 (d, J = 8.30 Hz, 2H, ArH), 7.35 (d, J = 8.90 Hz, 1H, ArH), 7.59 (d, J = 8.90 Hz, 1H, ArH), 7.64 (d, J = 8.40 Hz, 2H, ArH) ppm; ^{13}C NMR (125 MHz, DMSO-d_6) δ: 21.38, 29.40, 113.41, 115.54, 121.19, 124.32, 125.32, 129.19, 130.27, 131.56, 131.87, 135.28, 139.48, 149.33, 159.37, 163.12, 209.20 ppm. Anal. Calcd for $C_{19}H_{16}ClN_3O_2$ (353.81): C, 64.50; H, 4.56; N, 11.88; Found: C, 65.12; H, 4.87; N, 11.29 %.

*2.2.1.3. 4-Amino-6-chloro-2-(4-chlorobenzoyl)-N-methylquinoline-3-carboxamide **(4c)**.*

Yellow oil; FT-IR (KBr, cm^{-1}): 3452 and 3258 (NH_2), 3185 (NH), 1706 and 1681 (C═O), 1616 and 1489 (C═C aromatic groups); ^{1}H NMR (500 MHz, DMSO-d_6) δ: 2.72 (d, J = 4.72 Hz, 3H, CH_3), 7.03 (d, J = 4.72 Hz, 1H, NH), 7.31 (s, 2H, NH_2), 7.35 (dd, ^{1}J = 6.65, ^{2}J = 2.25 Hz, 2H, ArH), 7.59 (d, J = 7.65 Hz, 1H, ArH), 7.60 (d, J = 7.85 Hz, 1H, ArH), 7.73 (dd, ^{1}J = 6.55, ^{2}J = 2.15 Hz, 2H, ArH), 8.26 (d, J = 1.95 Hz, 1H, ArH) ppm; ^{13}C NMR (125 MHz, DMSO-d_6) δ: 29.43, 115.19, 120.54, 122.47, 125.82, 126.00, 127.16, 130.07, 132.77, 138.42, 141.75, 143.26, 147.62, 159.59, 162.88, 192.01 ppm. Calcd for $C_{18}H_{13}Cl_2N_3O_2$ (374.22): C, 57.77; H, 3.50; N, 11.23; Found: C, 58.39; H, 3.73; N, 10.74 %.

2.2.1.4. 4-Amino-2-benzoyl-6-chloro-N-methylquinoline-3-carboxamide ***(4d)***.

Yellow oil; FT-IR (KBr, cm^{-1}): 3448 and 3253 (NH_2), 3181 (NH), 1704 and 1679 (C═O), 1605 and 1492 (C═C aromatic groups); ^{1}H NMR (500 MHz, DMSO-d_6) δ: 2.73 (d, *J* = 4.67 Hz, 3H, CH_3), 6.99 (d, *J* = 4.67 Hz, 1H, NH), 7.25 (s, 2H, NH_2), 7.34 (d, *J* = 7.95 Hz, 2H, ArH), 7.41 (t, *J* = 8.90 Hz, 2H, ArH), 7.51 (t, *J* = 6.50 Hz, 1H, ArH), 7.59 (d, *J* = 8.80 Hz, 1H, 16ArH), 7.75 (d, *J* = 8.80 Hz, 1H, ArH), 8.26 (d, *J* = 1.90 Hz, 1H, ArH) ppm; ^{13}C NMR (125 MHz, DMSO-d_6) δ: 25.92, 115.10, 123.25, 123.79, 124.82, 125.21, 126.28, 129.82, 138.99, 144.20, 148.77, 156.10, 158.81, 159.67, 162.62, 192.64 ppm. Calcd for $C_{18}H_{14}ClN_3O_2$ (339.78): C, 63.63; H, 4.15; N, 12.37; Found: C, 64.27; H, 4.31; N, 12.03 %.

2.2.1.5. 4-Amino-2-(4-bromobenzoyl)-6-chloro-N-methylquinoline-3-carboxamide ***(4e)***.

Yellow oil; FT-IR (KBr, cm^{-1}): 3453 and 3259 (NH_2), 3187 (NH), 1705 and 1682 (C═O), 1608 and 1493 (C═C aromatic groups); ^{1}H NMR (500 MHz, DMSO-d_6) δ: 2.72 (d, *J* = 4.40 Hz, 3H, CH_3), 7.02 (d, *J* = 4.40 Hz, 1H, NH), 7.31 (s, 2H, NH_2), 7.35 (dd, ^{1}J = 8.90, ^{2}J = 2.30 Hz, 2H, ArH), 7.59 (dd, ^{1}J = 6.70, ^{2}J = 2.17 Hz, 2H, ArH), 7.66 (d, *J* = 8.85 Hz, 1H, ArH), 7.73 (d, *J* = 8.85 Hz, 1H, ArH), 8.26 (d, *J* = 1.95 Hz, 1H, ArH) ppm; ^{13}C NMR (125 MHz, DMSO-d_6) δ: 29.40, 115.43, 119.49, 121.20, 126.02, 127.61, 129.19, 129.67, 132.78, 137.60, 144.80, 156.65, 157.97, 158.41, 160.13, 194.90 ppm. Calcd for $C_{18}H_{13}BrClN_3O_2$ (418.68): C, 51.64; H, 3.13; N, 10.04; Found: C, 52.11; H, 3.19; N, 9.74 %.

2.2.1.6. 4-Amino-6-bromo-2-(4-bromobenzoyl)-N-methylquinoline-3-carboxamide ***(4f)***.

Yellow oil; FT-IR (KBr, cm^{-1}): 3449 and 3256 (NH_2), 3184 (NH), 1703 and 1679 (C═O), 1606 and 1490 (C═C aromatic groups); ^{1}H NMR (500 MHz, DMSO-d_6) δ: 2.72 (d, *J* = 4.65 Hz, 3H, CH_3), 7.02 (d, *J* = 4.65 Hz, 1H, NH), 7.31 (s, 2H, NH_2), 7.48 (dd, ^{1}J = 6.70, ^{2}J = 2.20 Hz, 2H, ArH), 7.54 (dd, ^{1}J = 6.70, ^{2}J = 2.17 Hz, 2H, ArH), 7.66 (d, *J* = 8.75 Hz, 1H, ArH), 7.73 (d, *J* = 8.70 Hz, 1H, ArH), 8.27 (d, *J* = 2.00 Hz, 1H, ArH) ppm; ^{13}C NMR (125 MHz, DMSO-d_6) δ: 29.41, 115.18, 119.84, 124.11, 126.01, 127.16, 132.10, 132.33, 132.56, 132.79, 138.01, 142.10, 150.47, 160.15, 162.91, 196.01 ppm. Calcd for $C_{18}H_{13}Br_2N_3O_2$ (463.13): C, 46.68; H, 2.83; N, 9.07; Found: C, 46.92; H, 2.88; N, 8.64 %.

2.2.1.7. 4-Amino-8-bromo-N-methyl-2-(4-methylbenzoyl)quinoline-3-carboxamide ***(4g)***.

Yellow oil; FT-IR (KBr, cm^{-1}): 3453 and 3259 (NH_2), 3186 (NH), 1704 and 1681 (C═O), 1614 and 1491 (C═C aromatic groups); ^{1}H NMR (500 MHz, DMSO-d_6) δ: 2.33 (s, 3H, CH_3), 2.73 (d, *J* = 4.97 Hz, 3H, CH_3), 6.92 (d, *J* = 4.97 Hz, 1H, NH), 7.17 (s, 2H, NH_2), 7.21 (d, *J* = 7.35 Hz, 1H, ArH), 7.23 (d, *J* = 8.30 Hz, 1H, ArH), 7.32 (d, *J* = 8.30 Hz, 2H, ArH), 7.38 (t, *J* = 7.65 Hz, 1H, ArH), 7.65 (d, *J* = 8.30 Hz, 2H, ArH) ppm; ^{13}C NMR (125 MHz, DMSO-d_6) δ: 21.37, 29.42, 115.55, 120.52, 124.32, 125.33, 128.61, 130.26, 136.71, 139.48, 142.40, 149.34, 153.46, 155.04, 159.35, 163.11, 197.11 ppm. Calcd for $C_{19}H_{16}BrN_3O_2$ (398.26): C, 57.30; H, 4.05; N, 10.55; Found: C, 56.71; H, 3.82; N, 10.69 %.

2.2.1.8. 4-Amino-6-chloro-2-(4-methoxybenzoyl)-N-methylquinoline-3-carboxamide ***(4h)***.

Yellow oil; FT-IR (KBr, cm^{-1}): 3453 and 3258 (NH_2), 3185 (NH), 1706 and 1681 (C═O), 1616 and 1494 (C═C aromatic groups); ^{1}H NMR (500 MHz, DMSO-d_6) δ: 2.72 (d, *J* = 4.65 Hz, 3H, CH_3), 3.79 (s, 3H, OCH_3), 6.91 (d, *J* = 4.65 Hz, 1H, NH), 6.94 (d, *J* = 8.70 Hz, 1H, ArH), 7.09 (dd, ^{1}J = 6.90, ^{2}J = 2.15 Hz, 2H, ArH), 7.14 (s, 2H, NH_2), 7.18 (s, 1H, ArH), 7.30 (d, *J* = 8.75 Hz, 1H, ArH), 7.69 (dd, ^{1}J = 6.85, ^{2}J = 2.07 Hz, 2H, ArH) ppm; ^{13}C NMR (125 MHz, DMSO-d_6) δ: 29.43, 55.85, 114.04, 115.28, 120.66, 126.15, 129.08, 131.64, 131.74, 132.90, 138.84, 145.32, 149.61, 159.14, 160.39, 163.18, 193.91 ppm. Calcd for $C_{19}H_{16}ClN_3O_3$ (369.81): C, 61.71; H, 4.36; N, 11.36; Found: C, 61.27; H, 4.28; N, 11.52 %.

2.2.1.9. 4-Amino-N,6-dimethyl-2-(4-methylbenzoyl)quinoline-3-carboxamide ***(4i)***.

Yellow oil; FT-IR (KBr, cm^{-1}): 3453 and 3259 (NH_2), 3180 (NH), 1707 and 1682 (C═O), 1617 and 1514 (C═C aromatic groups); ^{1}H NMR (500 MHz, DMSO-d_6) δ: 2.23 (s, 3H, CH_3), 2.33 (s, 3H, CH_3), 2.73 (d, *J* = 4.5 Hz, 3H, CH_3), 6.92 (s, 1H, NH), 7.09 (d, *J* = 8.6 Hz, 1H, ArH), 7.17 (s, 2H, NH_2), 7.22 (d, *J* = 7.40 Hz, 1H, ArH), 7.32 (d, *J* = 8.65 Hz, 2H, ArH), 7.44 (d, *J* = 8.55 Hz, 1H, ArH), 7.65 (d, *J* = 8.25 Hz, 2H, ArH) ppm; ^{13}C NMR (125 MHz, DMSO-d_6) δ: 21.30, 21.37, 29.47, 115.56, 119.58, 124.31, 125.32, 127.13, 129.24, 129.61, 130.25, 132.95, 136.20, 139.46, 149.32, 159.72, 163.13, 195.47 ppm. Calcd for $C_{20}H_{19}N_3O_2$ (333.39): C, 72.05; H, 5.74; N, 12.60; Found: C, 71.59; H, 5.57; N, 12.36 %.

4-Amino-2-(4-chlorobenzoyl)-6-methoxy-N-methylquinoline-3-carboxamide (4a).

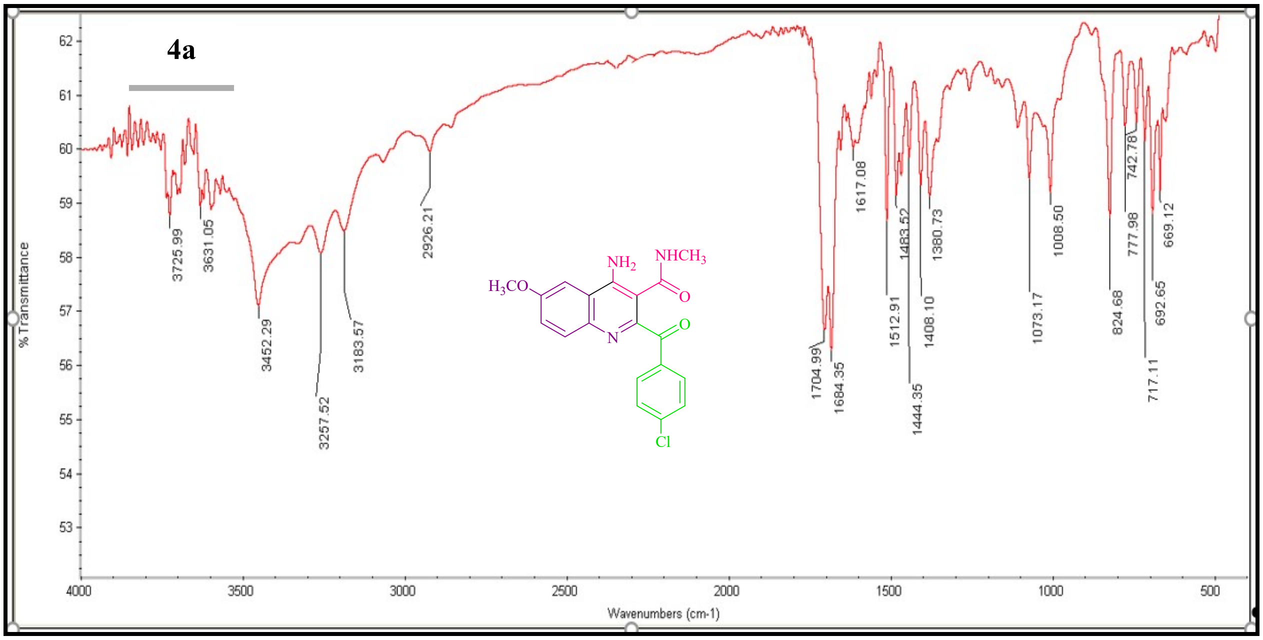

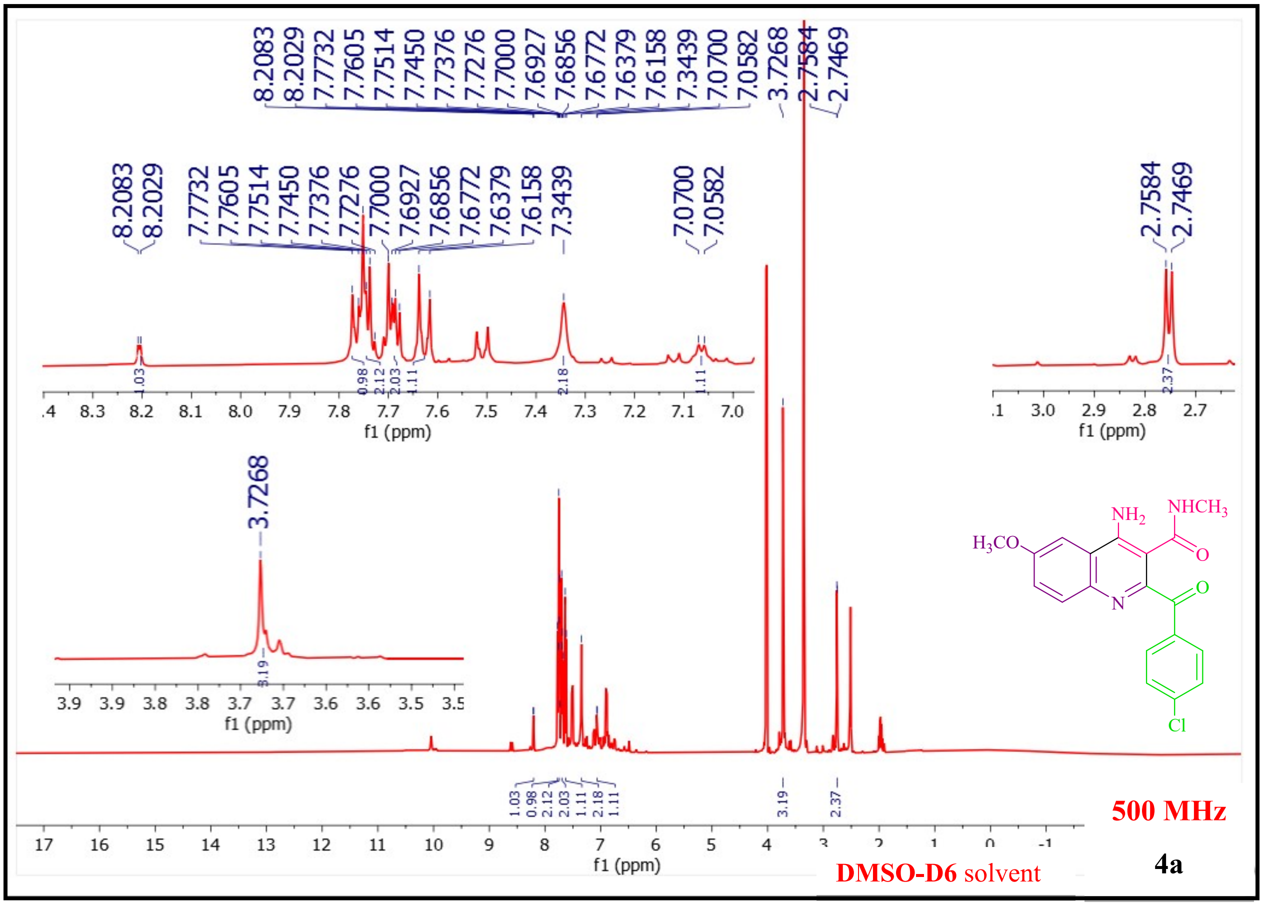

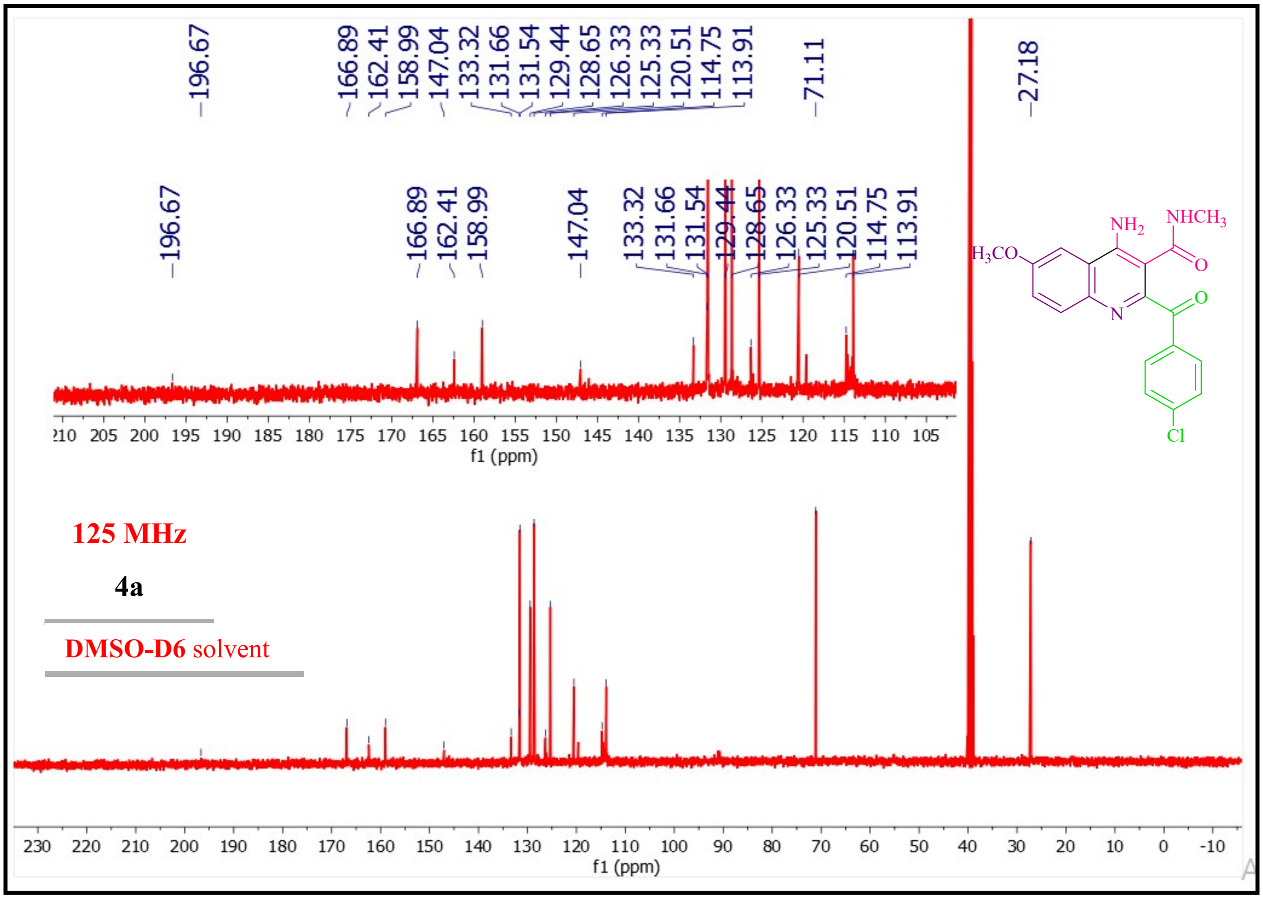

4-Amino-2-(4-chlorobenzoyl)-N,6-dimethylquinoline-3-carboxamide (4b).

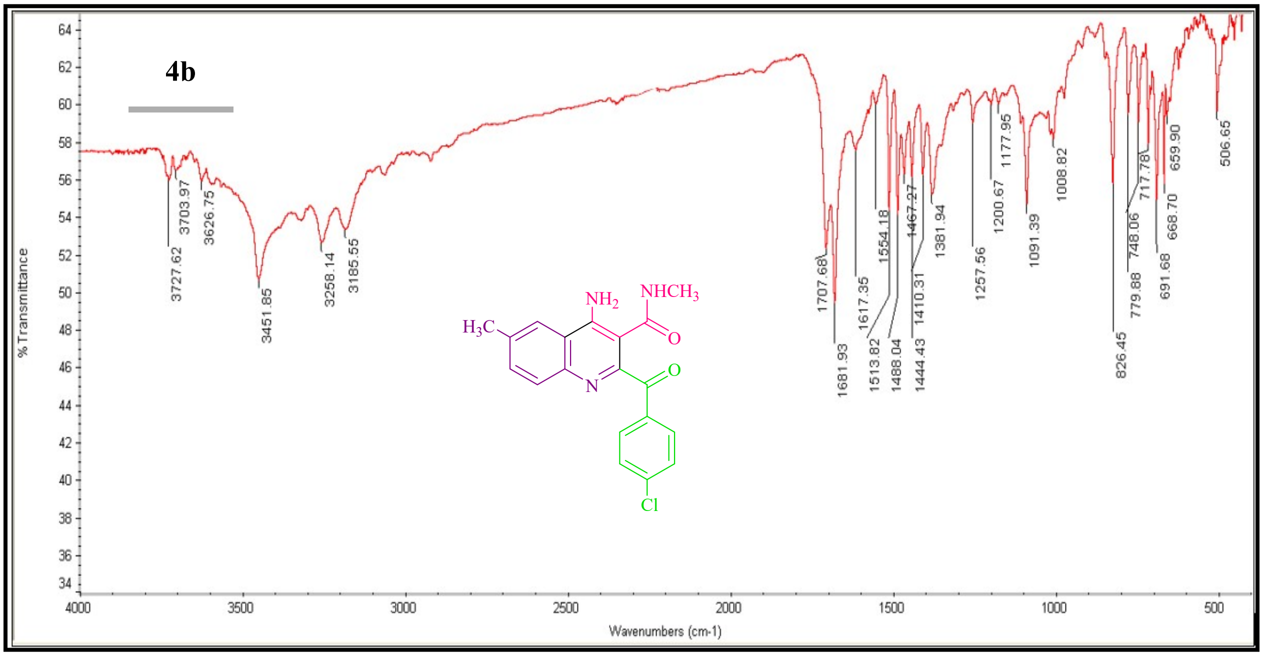

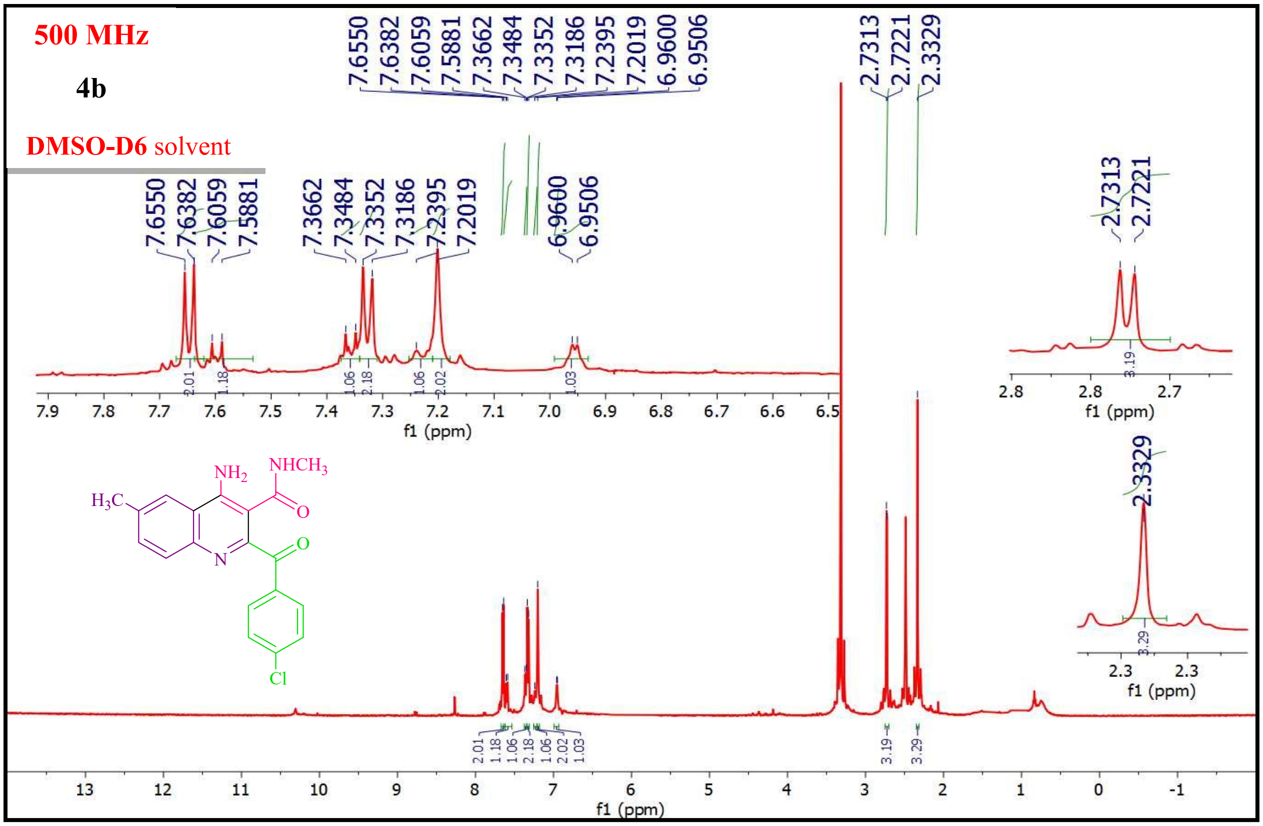

500 MHz
4b
DMSO-D6 solvent
7.6550
7.6382
7.6059
7.5881
7.3662
7.3484
7.3352
7.3186
7.2395
7.2019
6.9600
6.9506
2.7313
2.7221
2.3329
2.01
1.18
1.06
2.18
1.06
2.02
1.03
3.19
3.29
f1 (ppm)
NH2
NHCH3
H3C
O
N
Cl

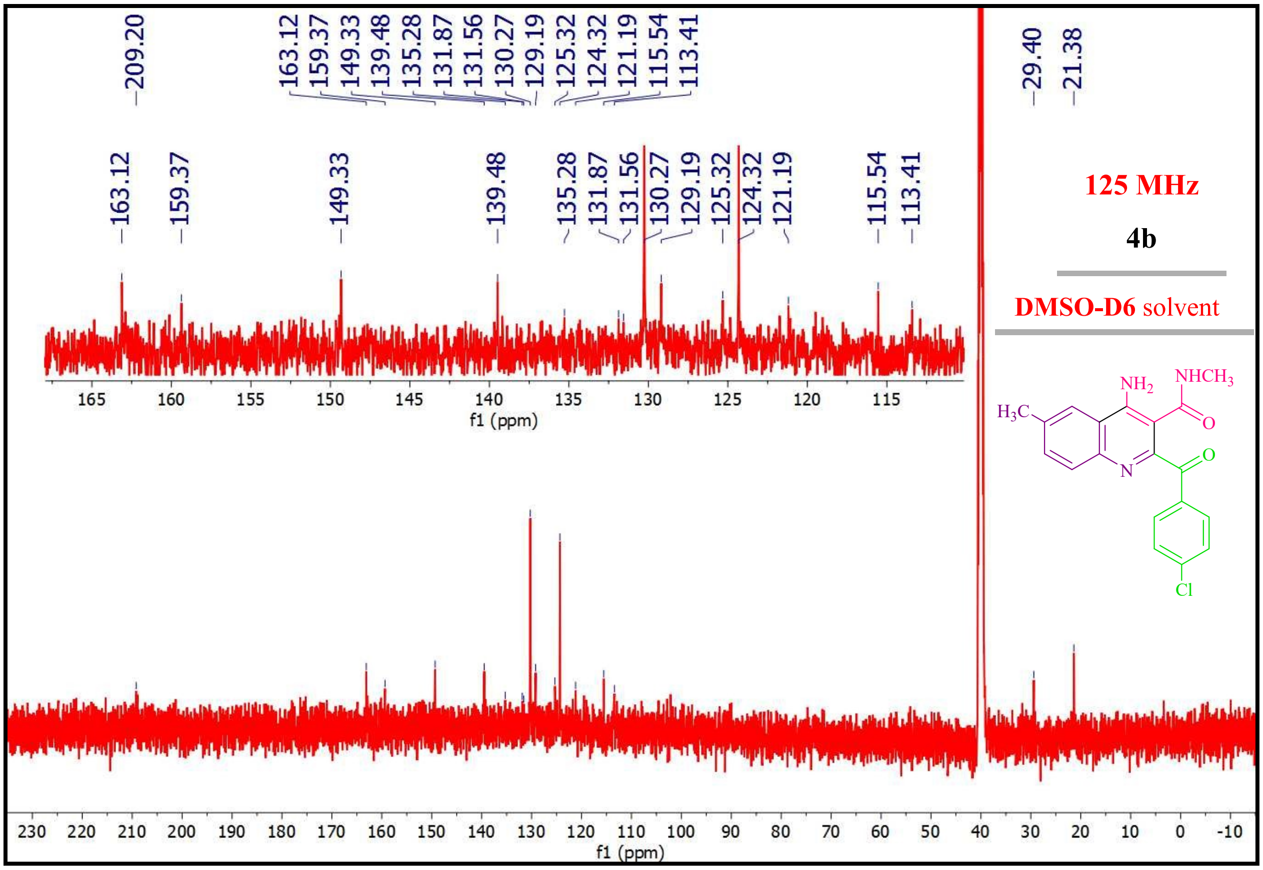

209.20
163.12
159.37
149.33
139.48
135.28
131.87
131.56
130.27
129.19
125.32
124.32
121.19
115.54
113.41
29.40
21.38
125 MHz
4b
DMSO-D6 solvent
NH2
NHCH3
H3C
O
N
O
Cl
f1 (ppm)

4-Amino-6-chloro-2-(4-chlorobenzoyl)-N-methylquinoline-3-carboxamide (4c).

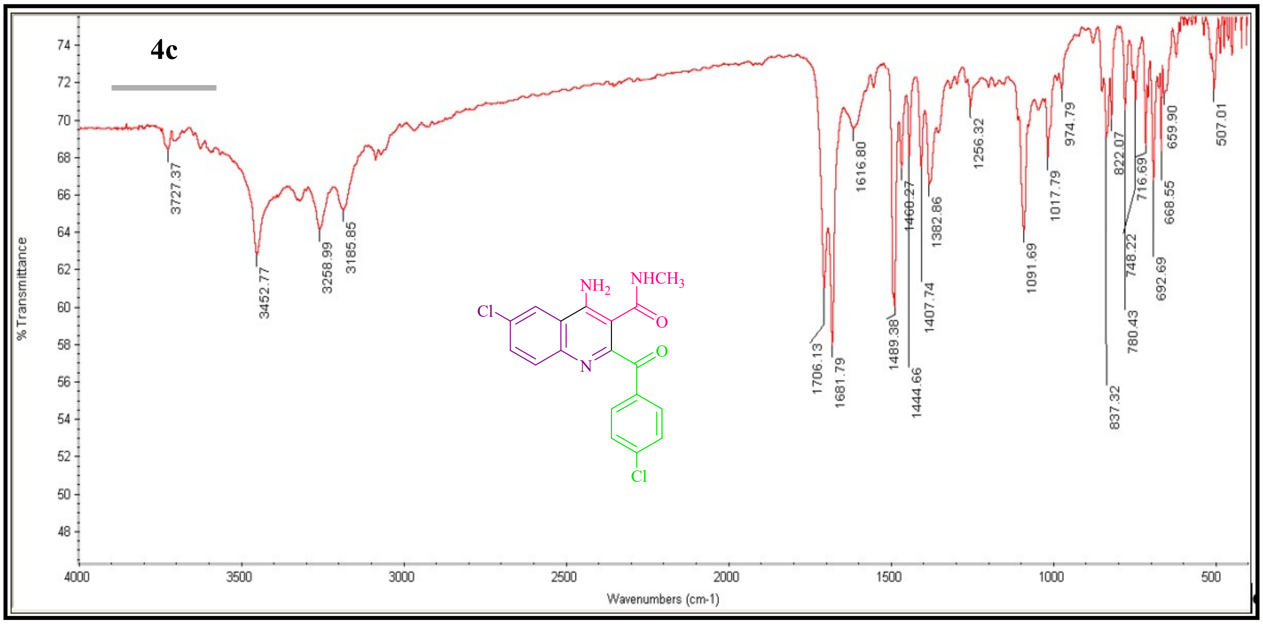

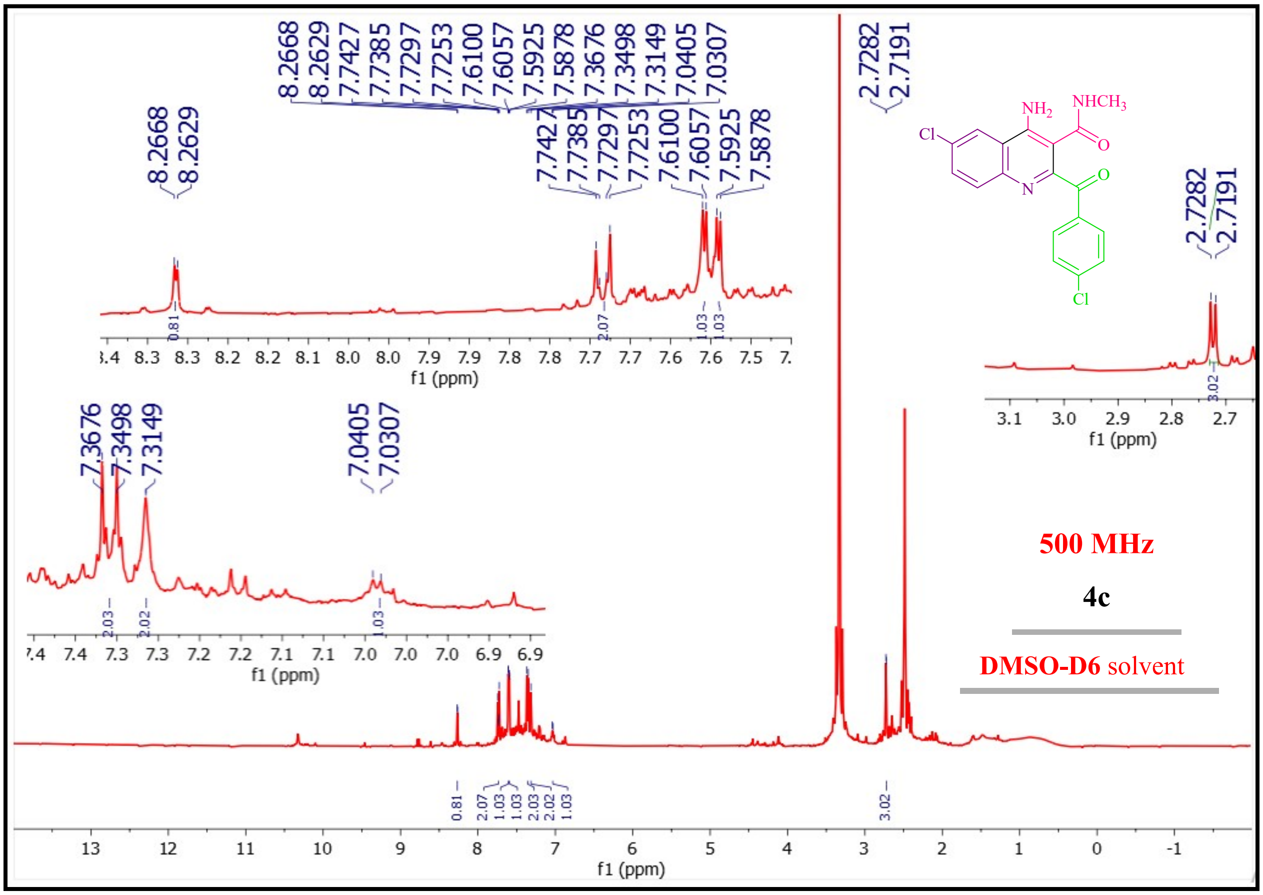

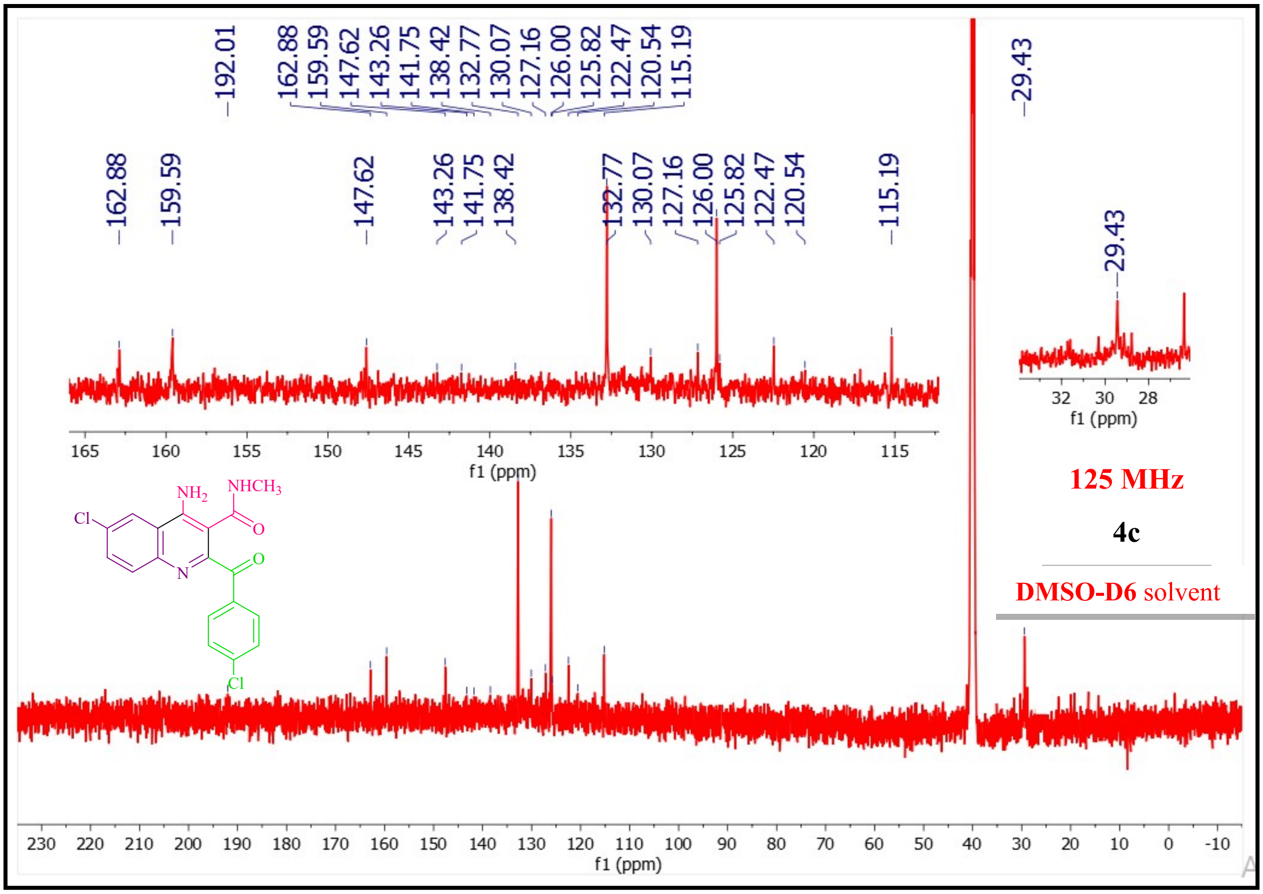

4-Amino-2-benzoyl-6-chloro-N-methylquinoline-3-carboxamide (4d).

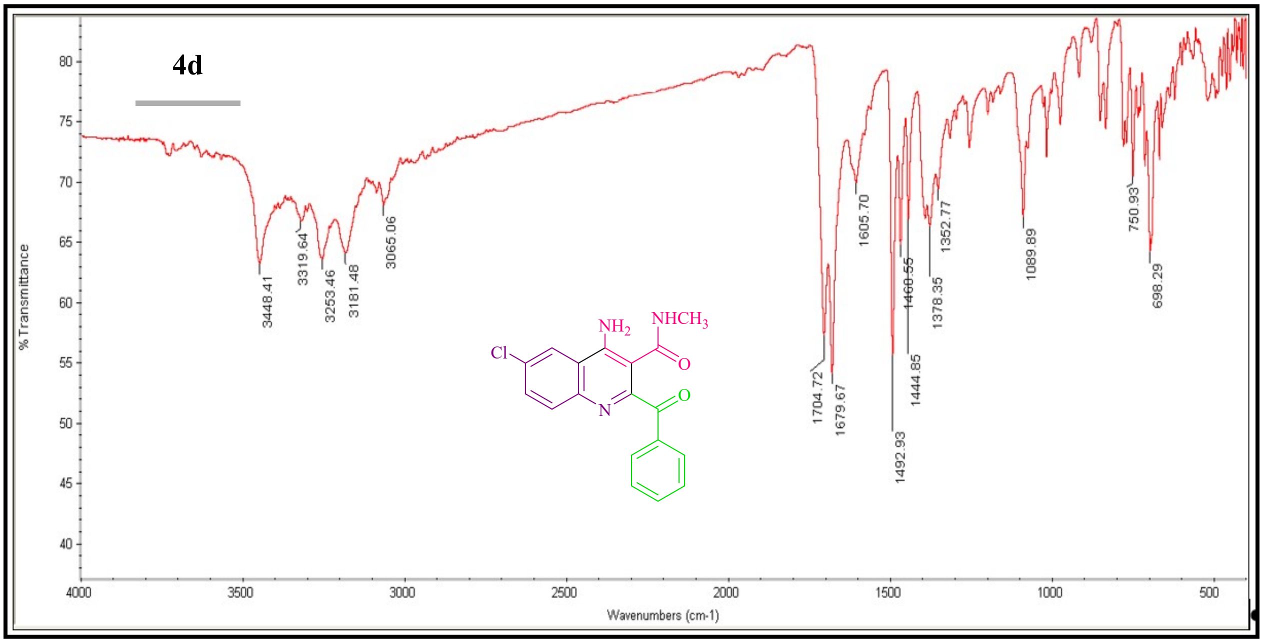

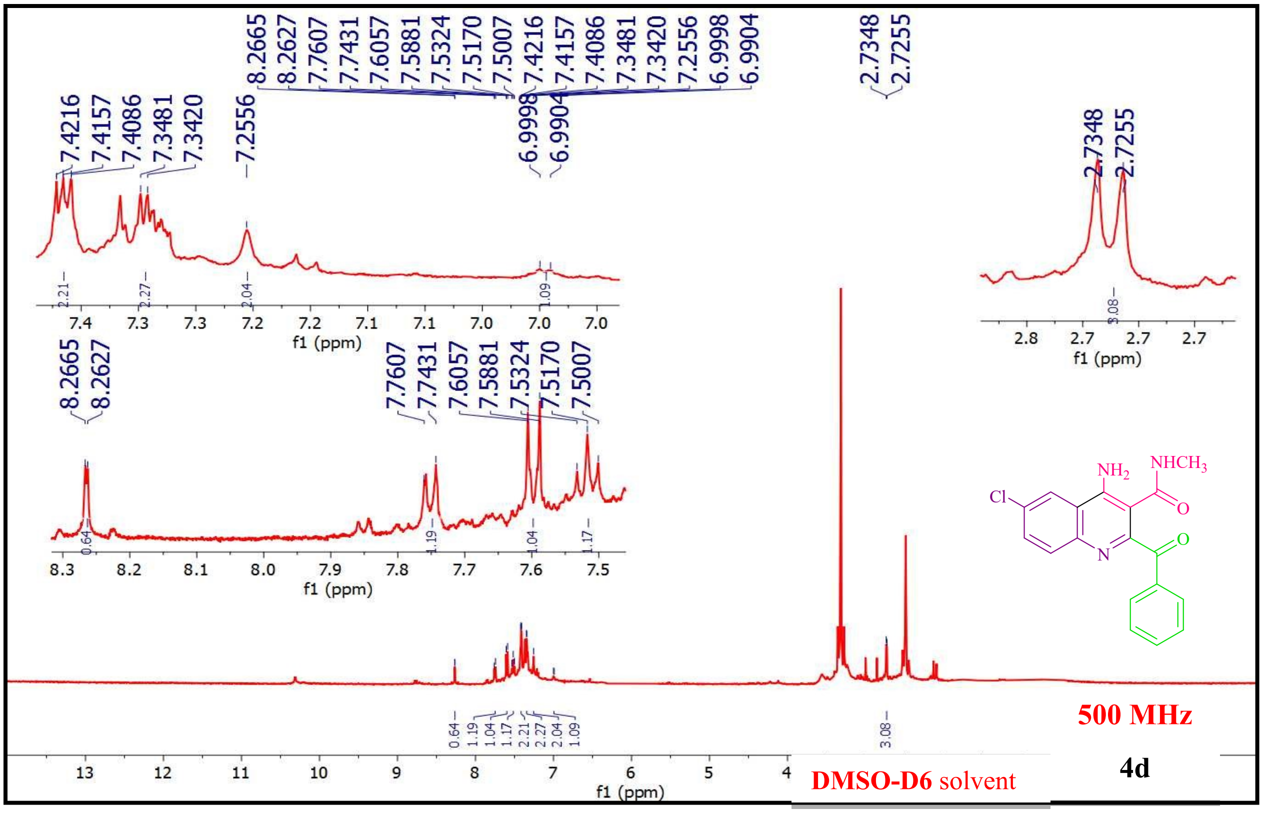

8.2665
8.2627
7.7607
7.7431
7.6057
7.5881
7.5324
7.5170
7.5007
7.4216
7.4157
7.4086
7.3481
7.3420
7.2556
6.9998
6.9904
2.7348
2.7255
f1 (ppm)
NH2
NHCH3
Cl
N
O
500 MHz
DMSO-D6 solvent
4d

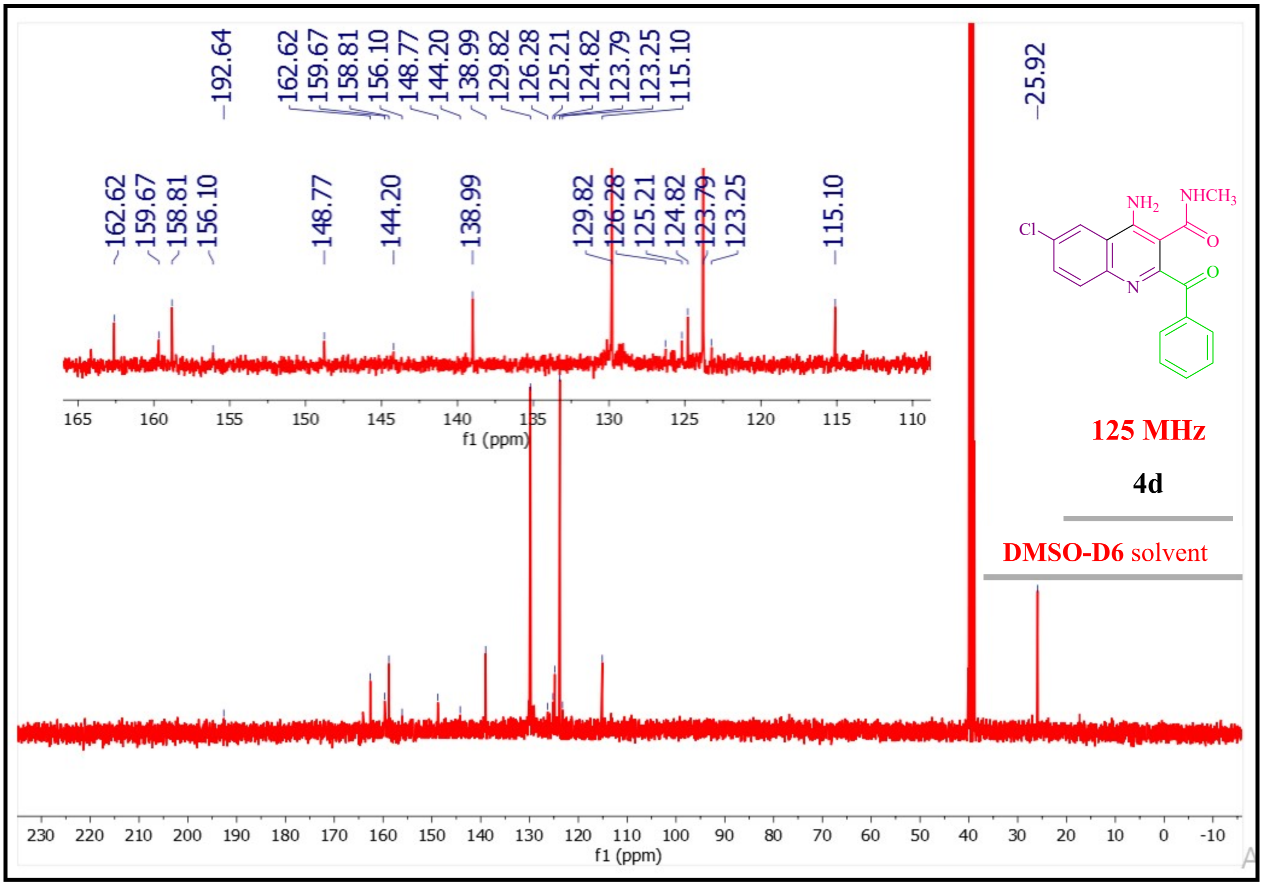

192.64
162.62
159.67
158.81
156.10
148.77
144.20
138.99
129.82
126.28
125.21
124.82
123.79
123.25
115.10
25.92
165 160 155 150 145 140 135 130 125 120 115 110
f1 (ppm)
NH2
NHCH3
Cl
N
O
125 MHz
4d
DMSO-D6 solvent
230 220 210 200 190 180 170 160 150 140 130 120 110 100 90 80 70 60 50 40 30 20 10 0 -10
f1 (ppm)

4-Amino-2-(4-bromobenzoyl)-6-chloro-N-methylquinoline-3-carboxamide (4e).

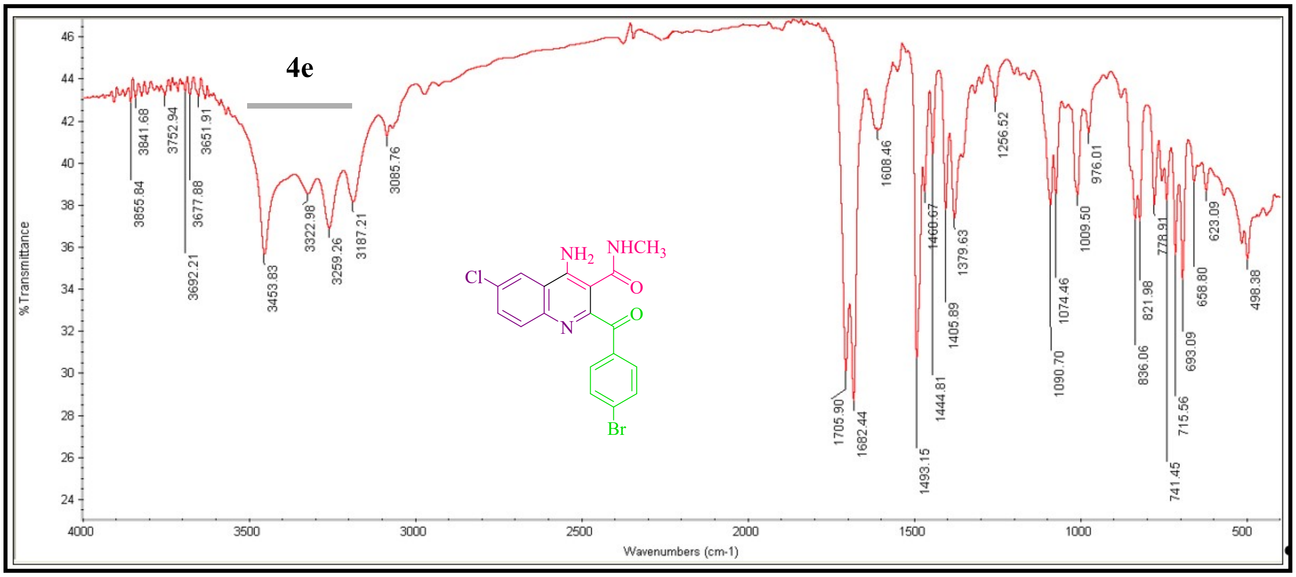

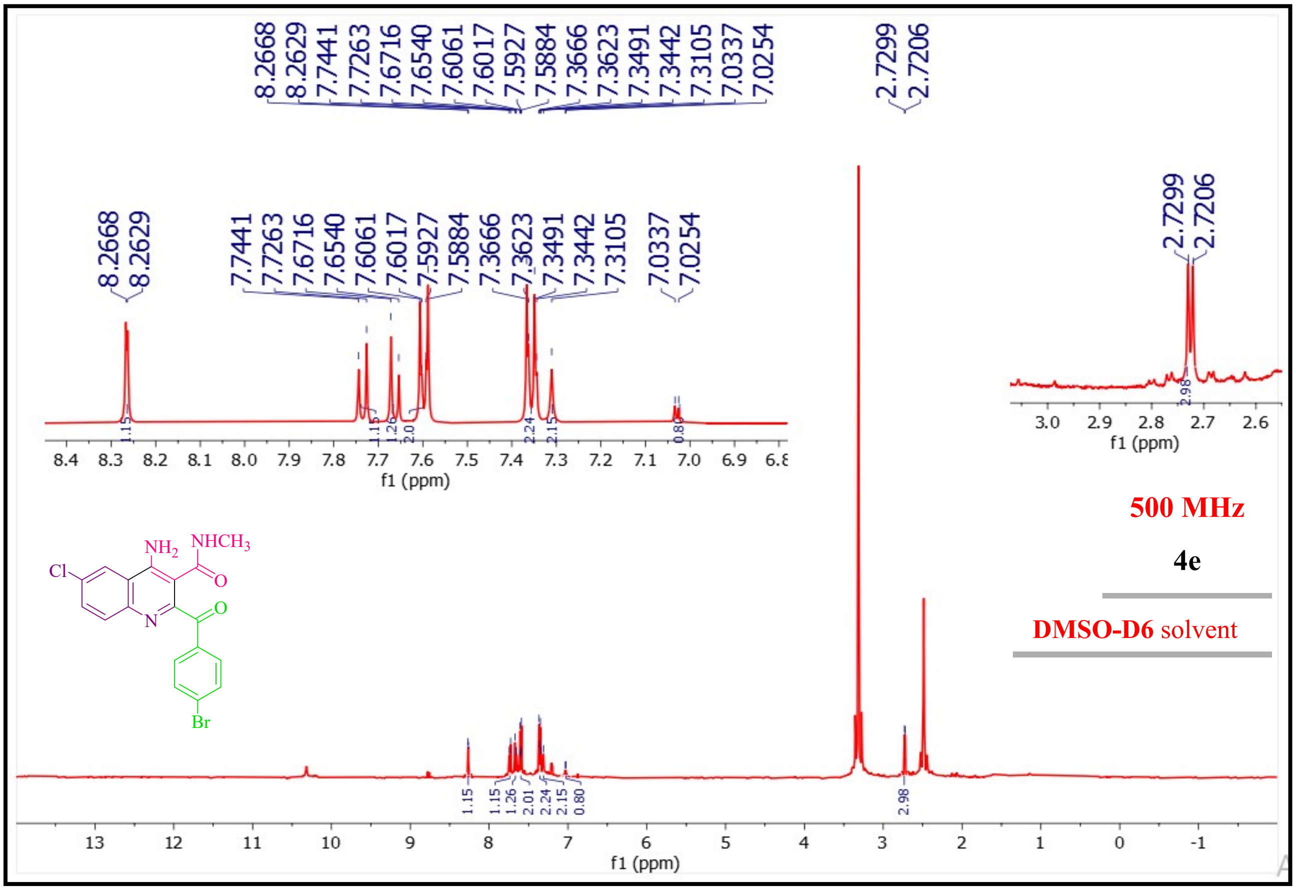

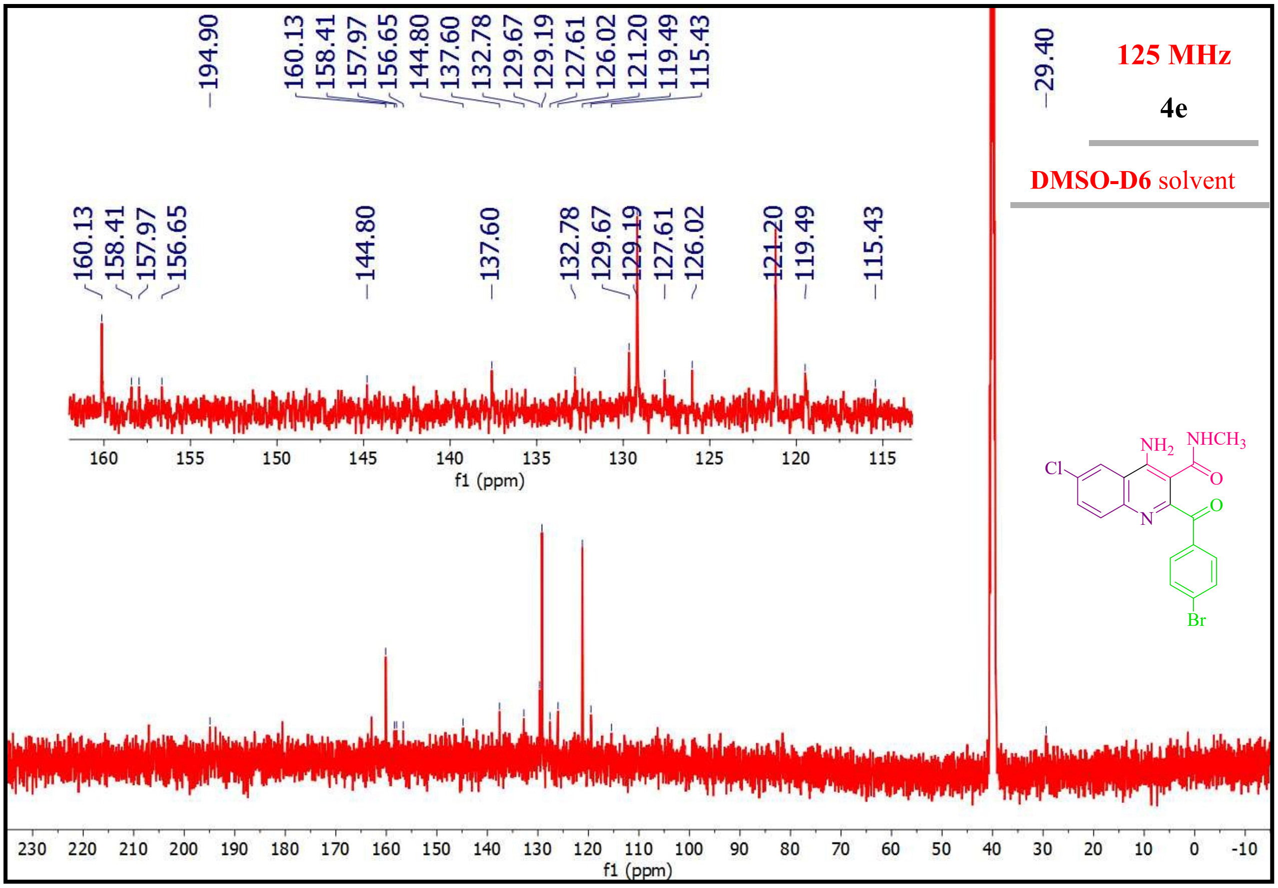

4-Amino-6-bromo-2-(4-bromobenzoyl)-N-methylquinoline-3-carboxamide (4f).

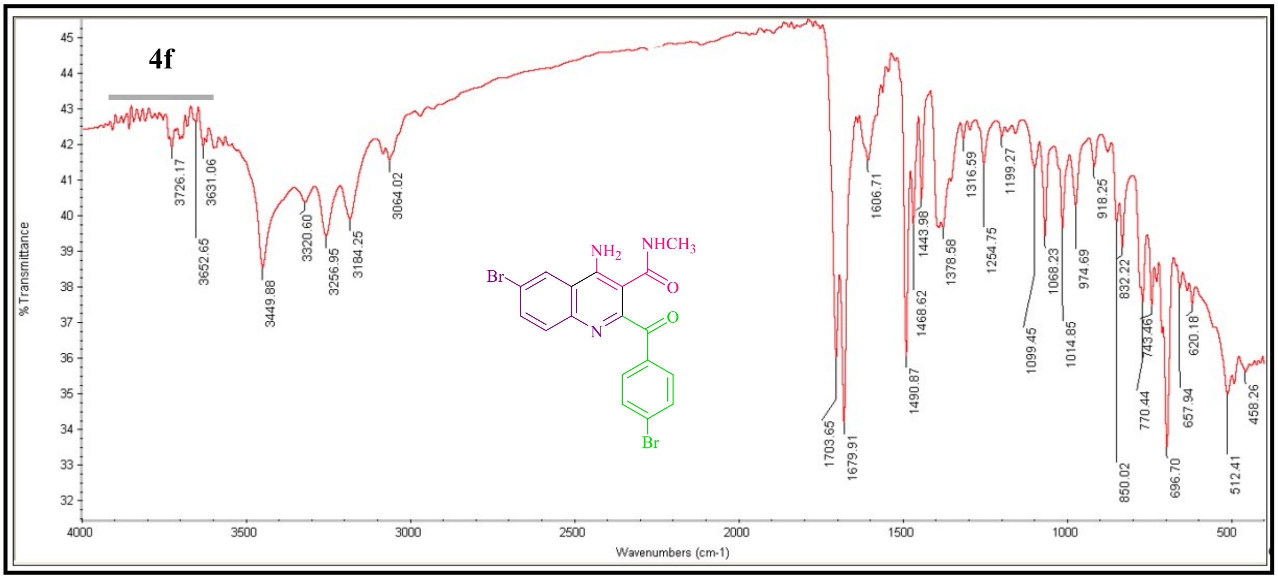

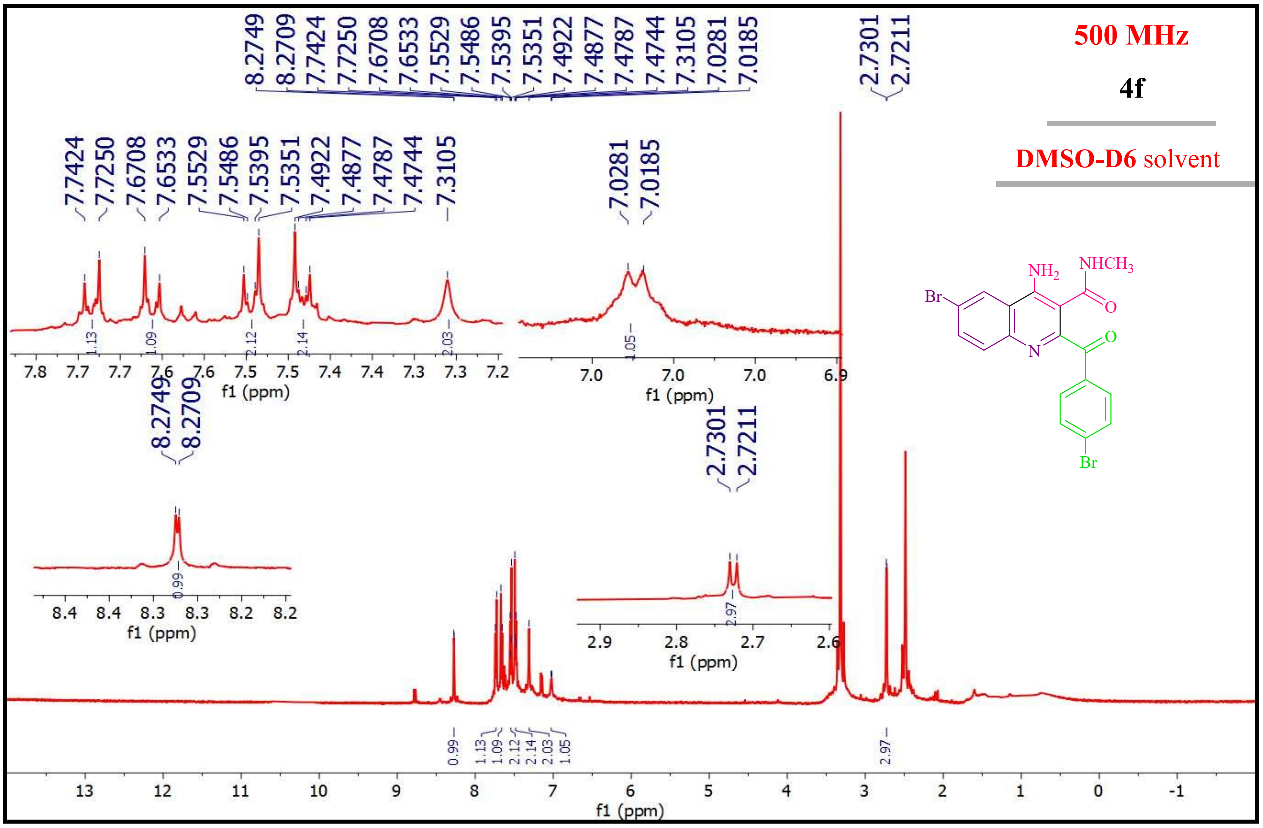
500 MHz
4f
DMSO-D6 solvent
8.2749 8.2709 7.7424 7.7250 7.6708 7.6533 7.5529 7.5486 7.5395 7.5351 7.4922 7.4877 7.4787 7.4744 7.3105 7.0281 7.0185
2.7301 2.7211
NH_2
$NHCH_3$
O
Br
N
O
Br
f1 (ppm)

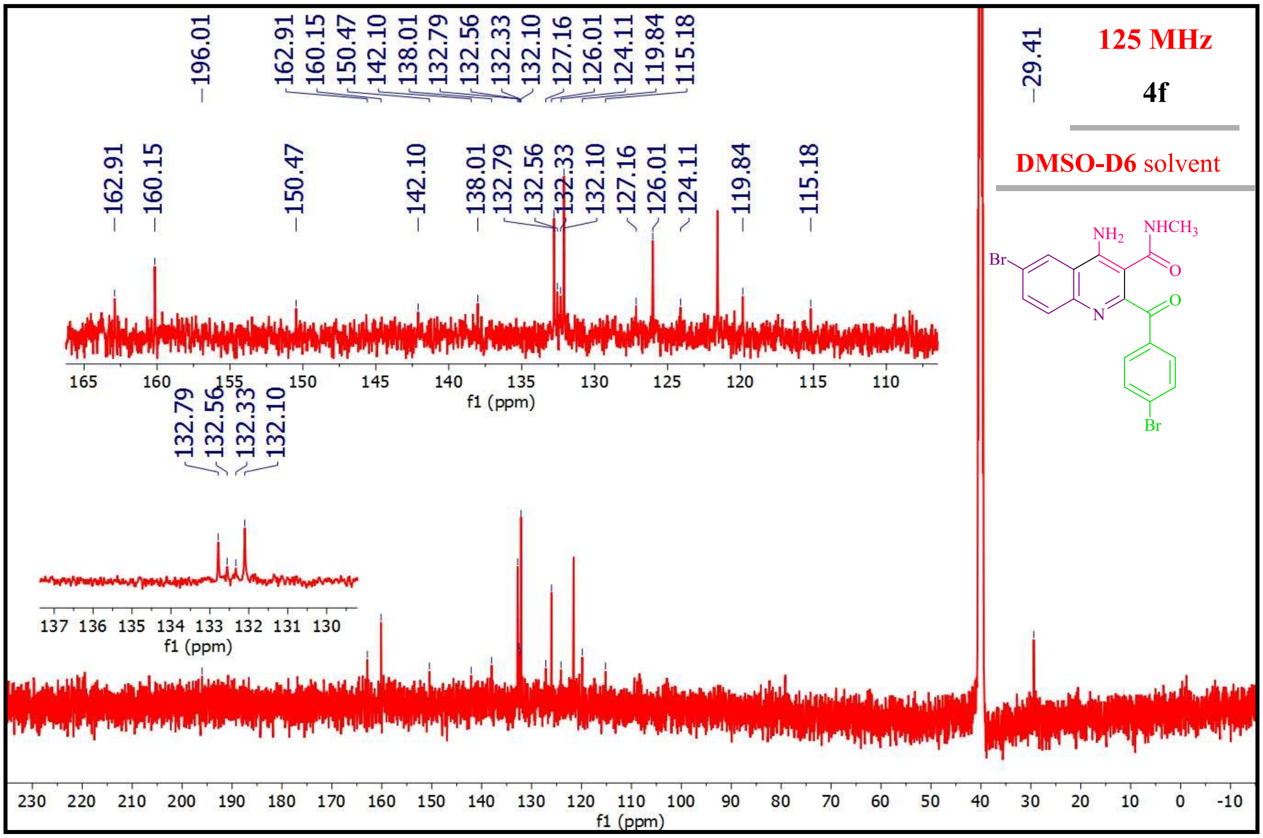
125 MHz
4f
DMSO-D6 solvent
196.01
162.91 160.15 150.47 142.10 138.01 132.79 132.56 132.33 132.10 127.16 126.01 124.11 119.84 115.18
29.41
NH_2
$NHCH_3$
O
Br
N
O
Br
f1 (ppm)

4-Amino-8-bromo-N-methyl-2-(4-methylbenzoyl)quinoline-3-carboxamide (4g).

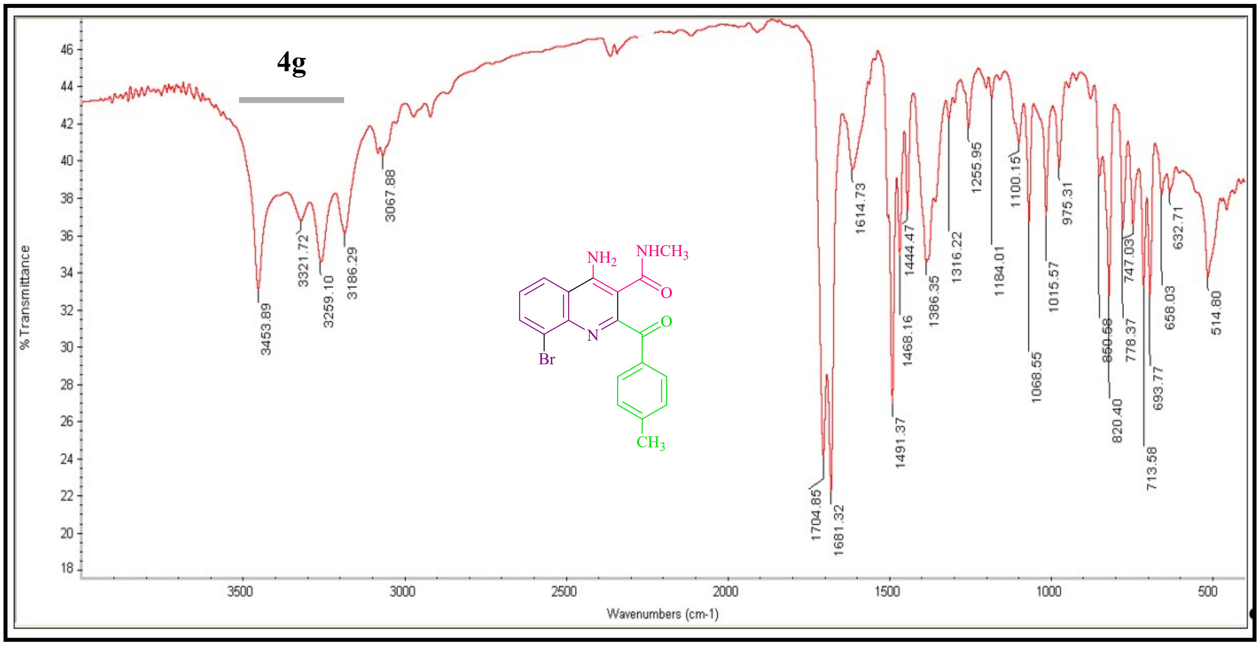

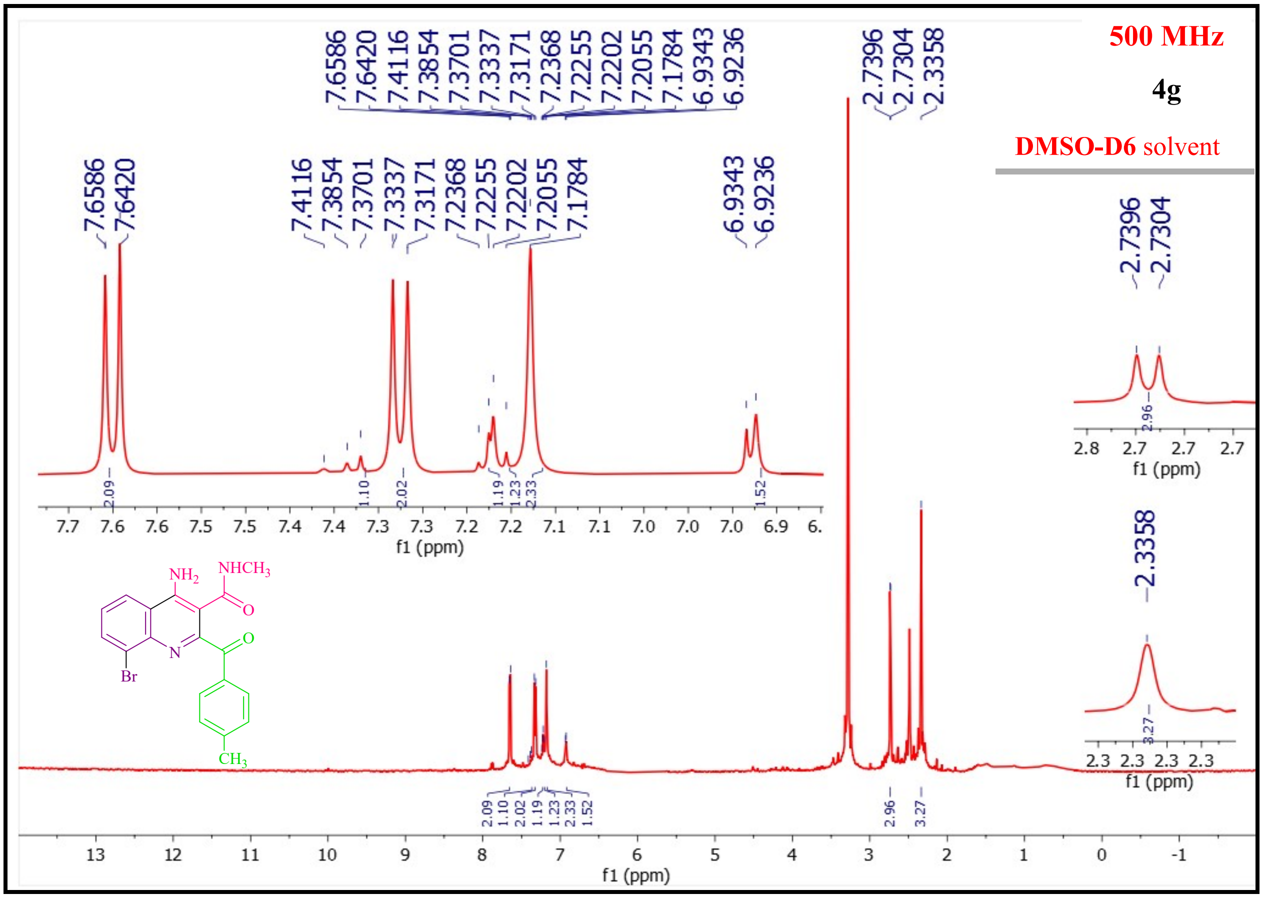

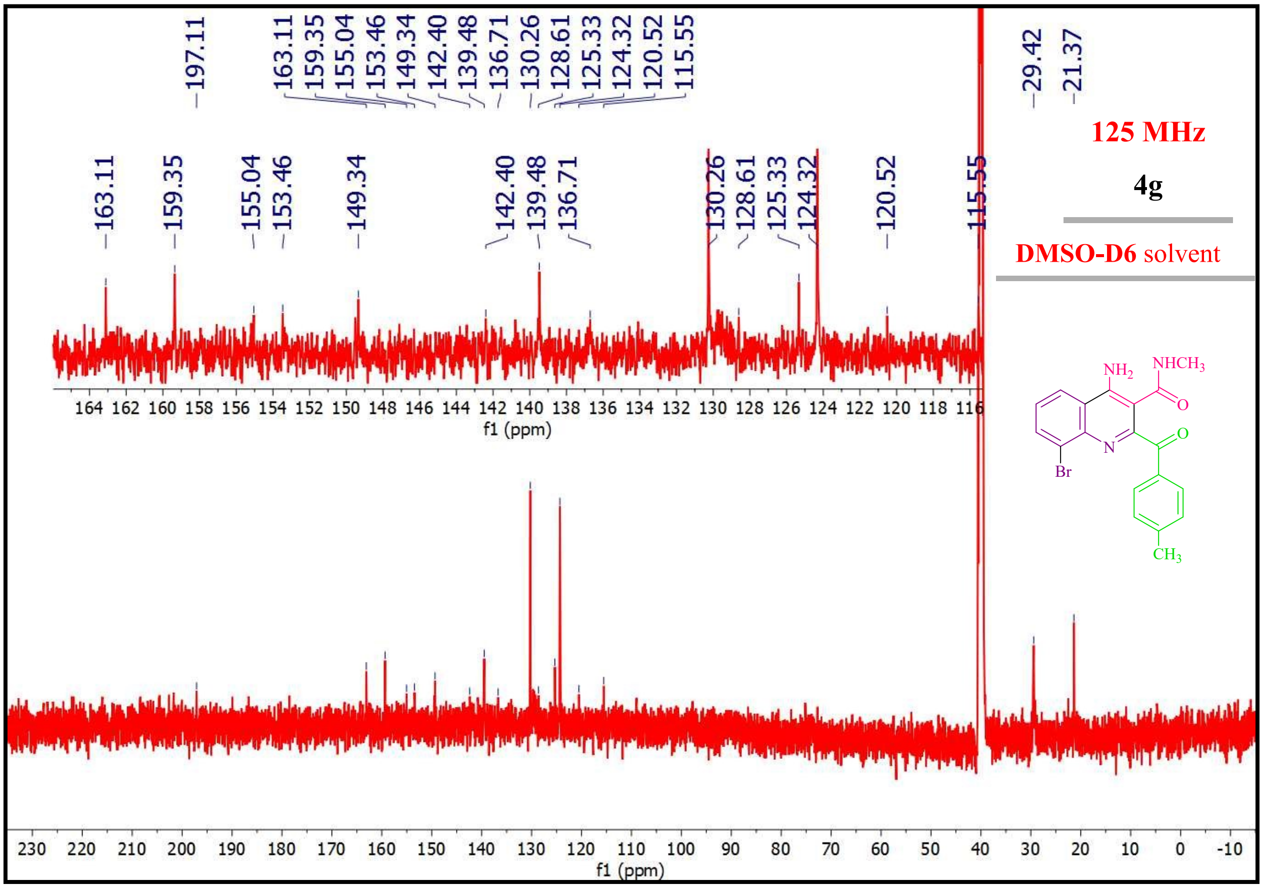

4-Amino-6-chloro-2-(4-methoxybenzoyl)-N-methylquinoline-3-carboxamide (4h).

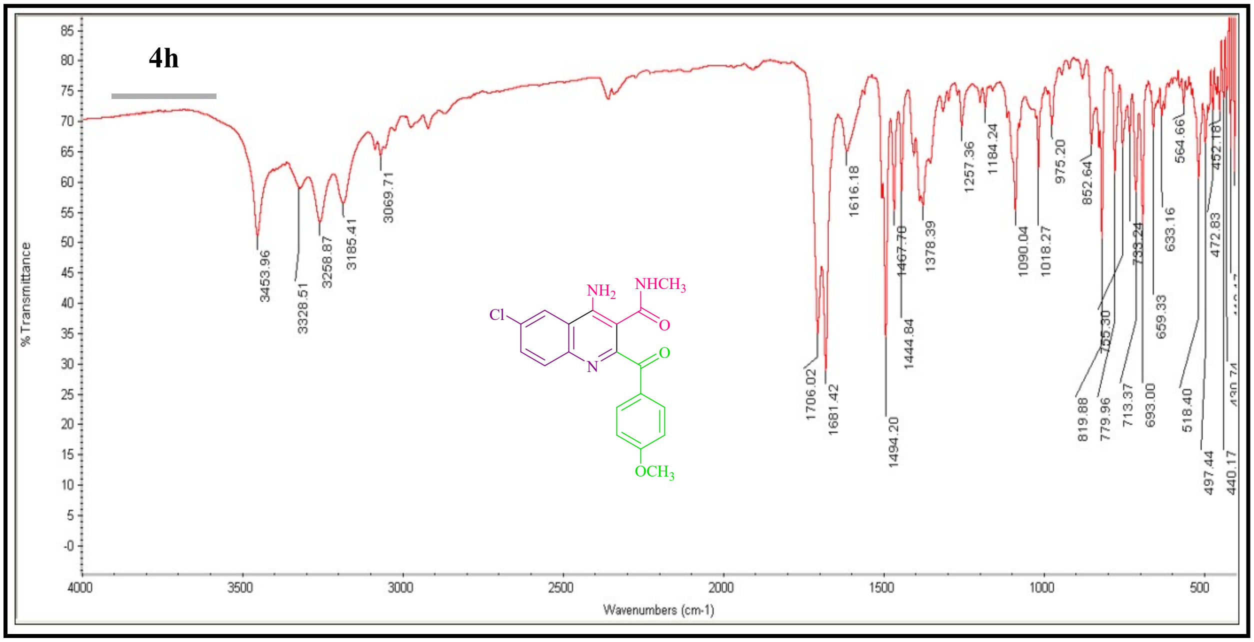

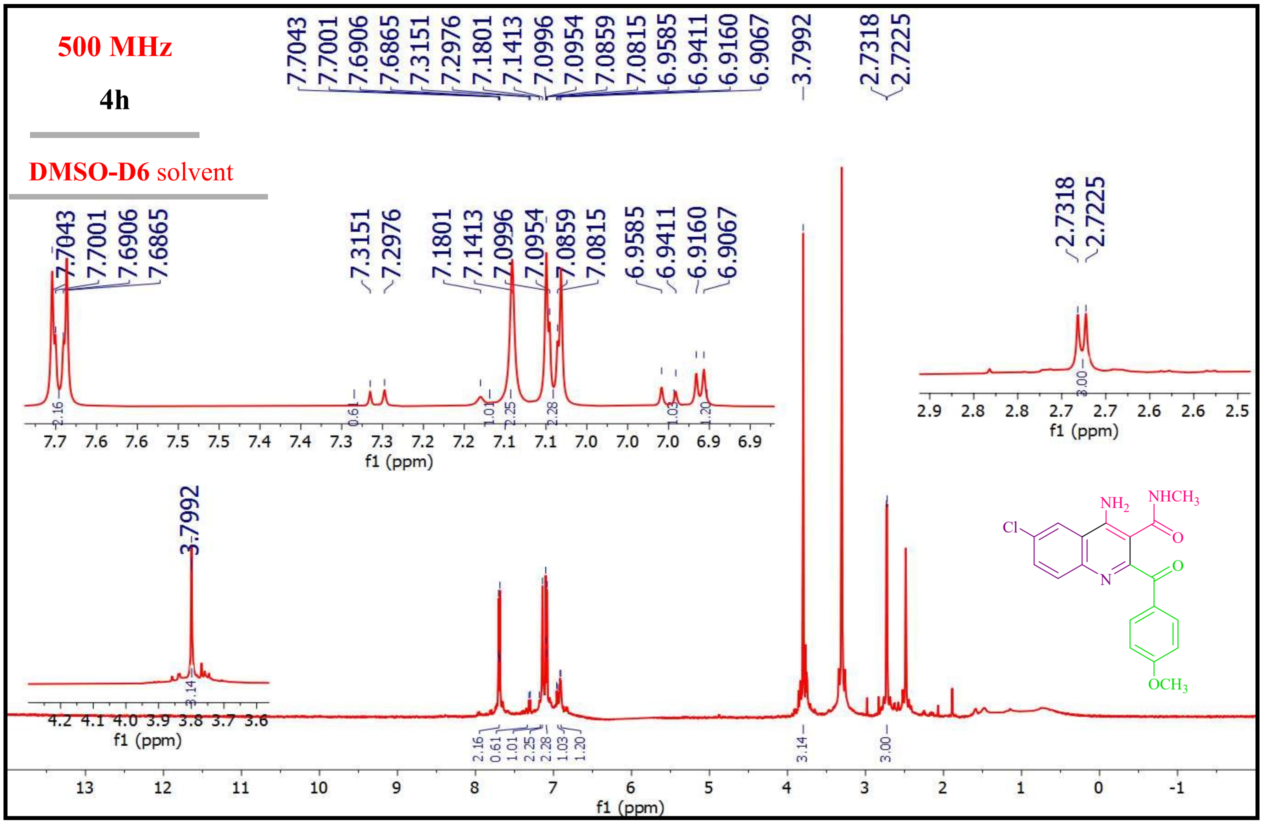
500 MHz
4h
DMSO-D6 solvent
7.7043
7.7001
7.6906
7.6865
7.3151
7.2976
7.1801
7.1413
7.0996
7.0954
7.0859
7.0815
6.9585
6.9411
6.9160
6.9067
3.7992
2.7318
2.7225
f1 (ppm)
NH2
NHCH3
Cl
N
O
OCH3

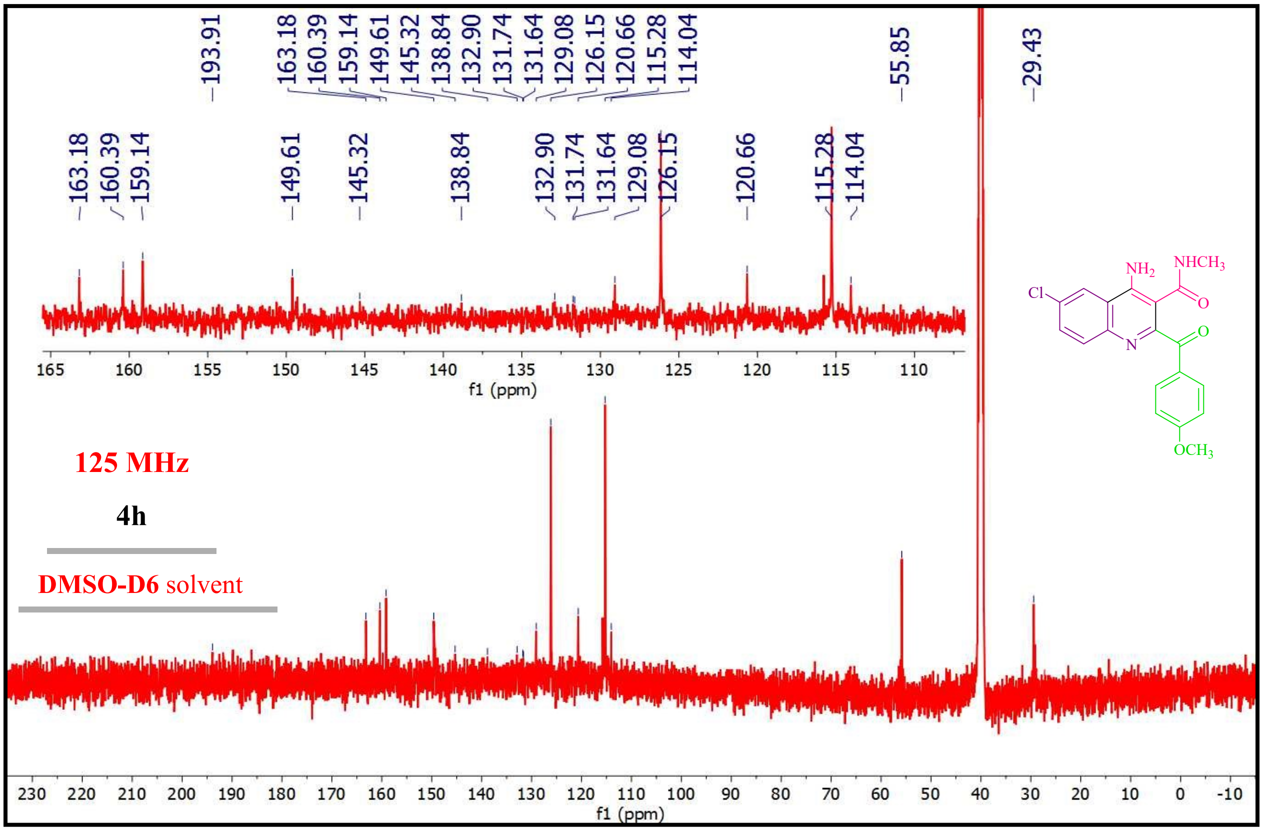
193.91
163.18
160.39
159.14
149.61
145.32
138.84
132.90
131.74
131.64
129.08
126.15
120.66
115.28
114.04
55.85
29.43
125 MHz
4h
DMSO-D6 solvent
f1 (ppm)
NH2
NHCH3
Cl
N
O
OCH3

4-Amino-N,6-dimethyl-2-(4-methylbenzoyl)quinoline-3-carboxamide (4i).

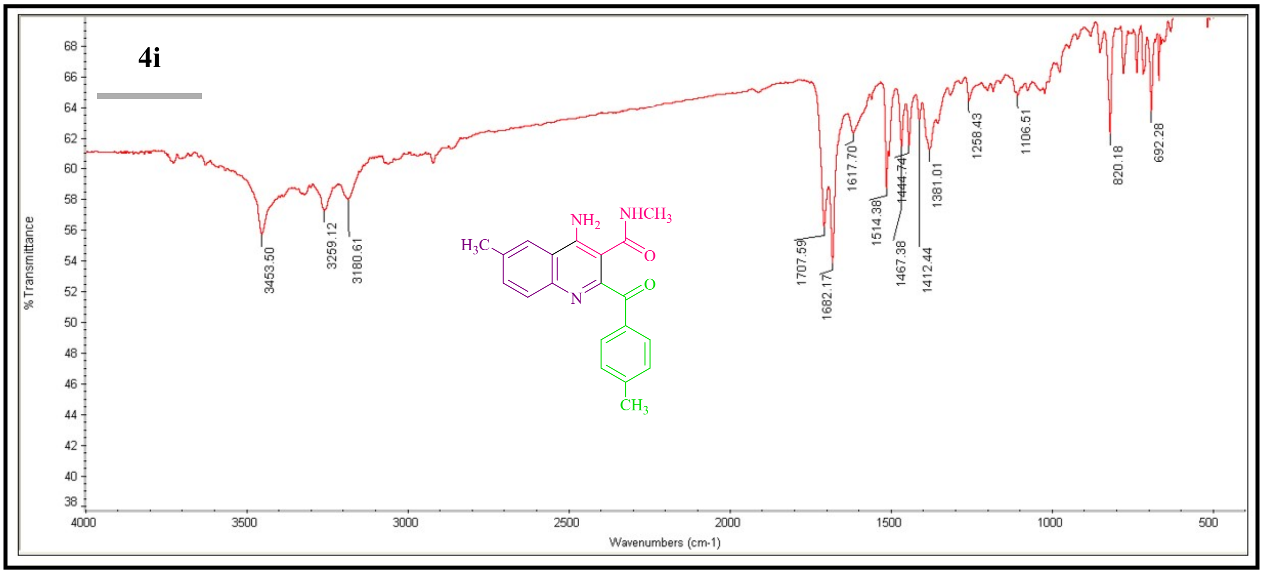

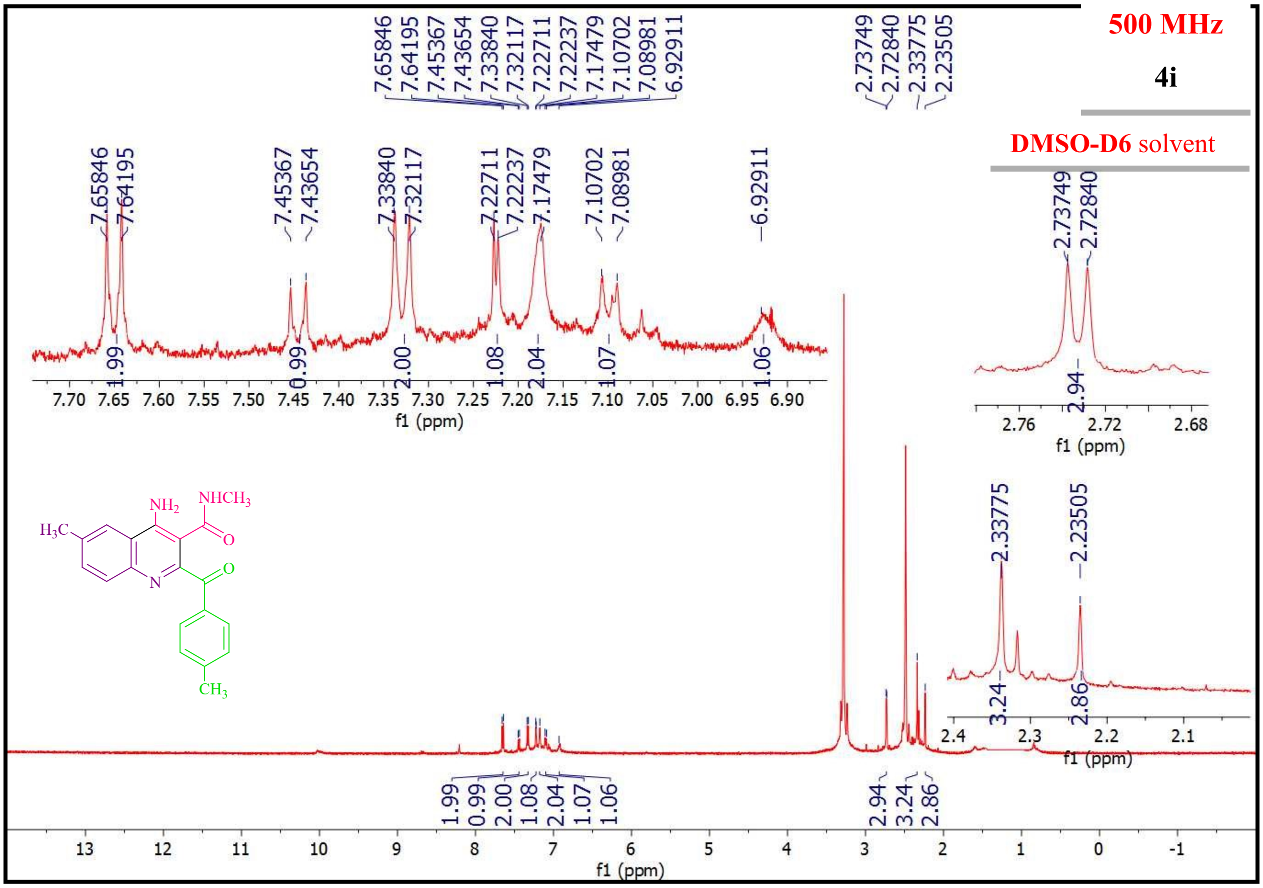

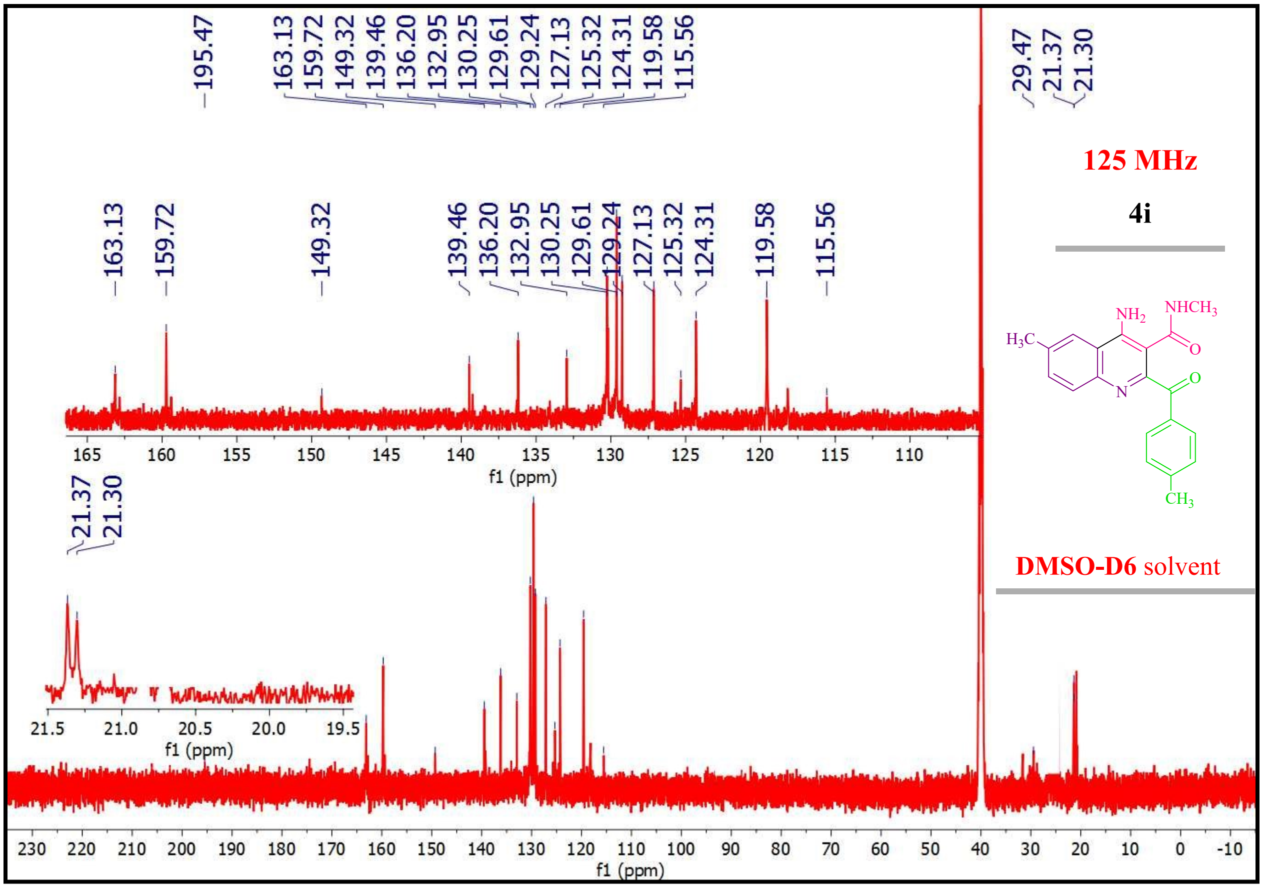

195.47
163.13
159.72
149.32
139.46
136.20
132.95
130.25
129.61
129.24
127.13
125.32
124.31
119.58
115.56
29.47
21.37
21.30
125 MHz
4i
NH2
NHCH3
H3C
O
N
CH3
DMSO-D6 solvent
f1 (ppm)

Table S1. The ΔE values of compounds (kJ/mol) obtained at B3LYP/6-311++G(d,p) level of theory.

Compound	Solvent					
	Solvent-free	H_2O	EtOH	CH_3CN	THF	DMF
4-a	-46.45	-63.15	-61.77	-62.36	-58.05	-62.42
4-b	-43.53	-62.16	-60.56	-61.25	-56.27	-61.32
4-c	-37.19	-52.05	-50.64	-51.24	-46.95	-51.30
4-d	-35.87	-49.72	-48.39	-48.96	-46.37	-49.02
4-e	-37.55	-52.38	-50.99	-51.59	-47.30	-51.65
4-f	-36.67	-51.38	-49.99	-50.58	-46.33	-50.64
4-g	-19.28	-43.82	-41.73	-42.63	-37.55	-42.71
4-h	-31.23	-46.15	-44.75	-45.35	-42.25	-45.41
4-i	-39.38	-58.27	-56.68	-57.36	-53.82	-57.43

Table S2. The ΔE_{ZPE} values of compounds (kJ/mol) obtained at B3LYP/6-311++G(d,p) level of theory.

Compound	Solvent					
	Solvent-free	H_2O	EtOH	CH_3CN	THF	DMF
4-a	-67.98	-85.17	-83.68	-84.31	-79.72	-84.38
4-b	-64.55	-83.65	-82.14	-82.79	-77.81	-82.87
4-c	-59.37	-74.81	-73.35	-73.99	-69.44	-74.05
4-d	-58.02	-71.99	-70.67	-71.24	-68.45	-71.30
4-e	-59.52	-74.68	-73.34	-73.92	-69.60	-73.98
4-f	-58.53	-73.83	-72.39	-73.01	-68.63	-73.08
4-g	-41.59	-66.74	-64.61	-65.24	-59.71	-65.61
4-h	-53.56	-69.22	-67.79	-68.40	-64.67	-68.47
4-i	-60.12	-79.77	-78.12	-78.83	-74.81	-78.90

Table S3. The $\Delta E_{thermal}$ values of compounds (kJ/mol) obtained at B3LYP/6-311++G(d,p) level of theory.

Compound	Solvent					
	Solvent-free	H_2O	EtOH	CH_3CN	THF	DMF
4-a	-55.89	-72.95	-71.50	-72.12	-67.66	-72.18
4-b	-52.99	-71.85	-70.25	-70.93	-65.84	-71.01
4-c	-47.11	-62.31	-60.86	-61.49	-57.02	-61.55
4-d	-45.91	-62.02	-60.70	-61.27	-56.39	-61.32
4-e	-47.32	-62.31	-60.94	-61.53	-57.19	-61.58
4-f	-46.32	-61.34	-59.93	-60.54	-56.22	-60.59
4-g	-29.29	-58.45	-56.36	-54.74	-47.30	-57.34
4-h	-41.25	-58.78	-57.36	-57.96	-52.20	-58.03
4-i	-48.85	-70.15	-68.55	-69.23	-63.29	-69.30

Table S4. The ΔH values of compounds (kJ/mol) obtained at B3LYP/6-311++G(d,p) level of theory.

Compound	Solvent					
	Solvent-free	H_2O	EtOH	CH_3CN	THF	DMF
4-a	-55.89	-72.95	-71.50	-72.12	-67.66	-72.18
4-b	-52.99	-71.84	-70.24	-70.93	-65.84	-71.01
4-c	-47.11	-62.31	-60.86	-61.49	-57.02	-61.54
4-d	-45.91	-62.02	-60.69	-61.27	-56.39	-61.32
4-e	-47.32	-62.31	-60.93	-61.53	-57.20	-61.58
4-f	-46.32	-61.34	-59.92	-60.54	-56.22	-60.60
4-g	-29.29	-58.45	-56.35	-54.74	-47.30	-57.34
4-h	-41.25	-58.78	-57.35	-57.97	-52.20	-58.03
4-i	-48.86	-70.15	-68.54	-69.23	-63.29	-69.30

Table S5. The ΔG values of compounds (kJ/mol) obtained at B3LYP/6-311++G(d,p) level of theory.

Compound	Solvent					
	Solvent-free	H_2O	EtOH	CH_3CN	THF	DMF
4-a	-27.47	-42.46	-41.03	-41.63	-37.10	-41.71
4-b	-18.34	-38.25	-37.65	-37.88	-35.03	-37.93
4-c	-19.30	-32.87	-31.65	-32.17	-28.31	-32.22
4-d	-17.58	-26.33	-25.23	-25.73	-26.09	-25.77
4-e	-18.75	-32.14	-31.25	-31.62	-28.31	-31.68
4-f	-16.94	-30.95	-29.69	-30.22	-26.48	-30.30
4-g	3.55	-18.02	-15.95	-20.77	-16.47	-16.91
4-h	-13.75	-24.98	-23.80	-24.31	-23.95	-24.35
4-i	-8.49	-32.01	-30.28	-31.06	-28.28	-31.09

Table S6. The electronic properties of compounds in solvent-free conditions obtained at B3LYP/6-311++G(d,p) level of theory.

Compound	E_g (eV)	IE (eV)	EA (eV)	μ (eV)	η (eV)	σ (eV^{-1})	ω (eV)
4-a	4.147	6.140	1.993	–4.066	2.074	0.482	3.987
4-b	4.441	6.399	1.959	–4.179	2.220	0.450	3.933
4-c	4.369	6.625	2.256	–4.441	2.185	0.458	4.514
4-d	4.376	6.528	2.152	–4.340	2.188	0.457	4.304
4-e	4.369	6.627	2.258	–4.443	2.185	0.458	4.518
4-f	4.350	6.619	2.268	–4.443	2.175	0.460	4.539
4-g	4.300	6.395	2.095	–4.245	2.150	0.465	4.190
4-h	4.370	6.441	2.071	–4.256	2.185	0.458	4.144
4-i	4.452	6.260	1.808	–4.034	2.226	0.449	3.656

Table S7. The electronic properties of compounds in H_2O solvent obtained at B3LYP/6-311++G(d,p) level of theory.

Compound	E_g (eV)	IE (eV)	EA (eV)	μ (eV)	η (eV)	σ (eV^{-1})	ω (eV)
4-a	3.810	6.085	2.275	-4.180	1.905	0.525	4.586
4-b	4.014	6.300	2.285	-4.293	2.007	0.498	4.590
4-c	4.101	6.441	2.340	-4.391	2.051	0.488	4.701
4-d	4.183	6.430	2.247	-4.338	2.091	0.478	4.500
4-e	4.079	6.440	2.361	-4.400	2.039	0.490	4.747
4-f	4.086	6.439	2.354	-4.397	2.043	0.490	4.731
4-g	4.206	6.406	2.200	-4.303	2.103	0.475	4.402
4-h	4.279	6.397	2.118	-4.258	2.139	0.467	4.237
4-i	4.162	6.270	2.109	-4.189	2.081	0.481	4.217

Table S8. The electronic properties of compounds in CH_3CN solvent obtained at B3LYP/6-311++G(d,p) level of theory.

Compound	E_g (eV)	IE (eV)	EA (eV)	μ (eV)	η (eV)	σ (eV^{-1})	ω (eV)
4-a	3.829	6.087	2.258	-4.173	1.915	0.522	4.547
4-b	4.032	6.302	2.271	-4.286	2.016	0.496	4.557
4-c	4.121	6.449	2.327	-4.388	2.061	0.485	4.672
4-d	4.201	6.435	2.234	-4.334	2.101	0.476	4.471
4-e	4.097	6.446	2.349	-4.397	2.048	0.488	4.720
4-f	4.104	6.446	2.342	-4.394	2.052	0.487	4.705
4-g	4.222	6.409	2.186	-4.297	2.111	0.474	4.374
4-h	4.291	6.400	2.110	-4.255	2.145	0.466	4.220
4-i	4.179	6.270	2.091	-4.180	2.090	0.479	4.181

Table S9. The electronic properties of compounds in DMF solvent obtained at B3LYP/6-311++G(d,p) level of theory.

Compound	E_g (eV)	IE (eV)	EA (eV)	μ (eV)	η (eV)	σ (eV^{-1})	ω (eV)
4-a	3.828	6.087	2.259	-4.173	1.914	0.522	4.549
4-b	4.031	6.302	2.271	-4.286	2.015	0.496	4.558
4-c	4.120	6.448	2.328	-4.388	2.060	0.485	4.674
4-d	4.200	6.434	2.235	-4.335	2.100	0.476	4.474
4-e	4.096	6.446	2.350	-4.398	2.048	0.488	4.722
4-f	4.103	6.446	2.343	-4.395	2.051	0.487	4.707
4-g	4.221	6.408	2.187	-4.298	2.111	0.474	4.376
4-h	4.290	6.400	2.111	-4.256	2.145	0.466	4.222
4-i	4.178	6.270	2.092	-4.181	2.089	0.479	4.185

Table S10. The electronic properties of compounds in EtOH solvent obtained at B3LYP/6-311++G(d,p) level of theory.

	E_g (eV)	IE (eV)	EA (eV)	μ (eV)	η (eV)	σ (eV^{-1})	ω (eV)
4-a	3.844	6.089	2.245	-4.167	1.922	0.520	4.516
4-b	4.045	6.304	2.259	-4.281	2.023	0.494	4.531
4-c	4.137	6.454	2.317	-4.385	2.068	0.483	4.648
4-d	4.214	6.438	2.224	-4.331	2.107	0.475	4.451
4-e	4.111	6.451	2.340	-4.396	2.055	0.487	4.701
4-f	4.118	6.451	2.333	-4.392	2.059	0.486	4.684
4-g	4.234	6.410	2.176	-4.293	2.117	0.472	4.354
4-h	4.299	6.403	2.104	-4.253	2.149	0.465	4.208
4-i	4.193	6.271	2.078	-4.174	2.097	0.477	4.155

Table S11. The electronic properties of compounds in THF solvent obtained at B3LYP/6-311++G(d,p) level of theory.

Compound	E_g (eV)	IE (eV)	EA (eV)	μ (eV)	η (eV)	σ (eV^{-1})	ω (eV)
4-a	3.937	6.095	2.158	-4.127	1.968	0.508	4.325
4-b	4.173	6.324	2.152	-4.238	2.086	0.479	4.304
4-c	4.232	6.486	2.253	-4.370	2.116	0.473	4.511
4-d	4.295	6.459	2.164	-4.312	2.148	0.466	4.329
4-e	4.202	6.483	2.281	-4.382	2.101	0.476	4.571
4-f	4.209	6.483	2.274	-4.378	2.104	0.475	4.555
4-g	4.287	6.414	2.127	-4.270	2.143	0.467	4.254
4-h	4.330	6.412	2.082	-4.247	2.165	0.462	4.165
4-i	4.289	6.274	1.985	-4.129	2.145	0.466	3.976
